# A non-structural protein 1 substitution of dengue virus enhances viral replication by interfering with the antiviral signaling pathway

**DOI:** 10.1186/s12929-024-01116-4

**Published:** 2025-02-20

**Authors:** Jing-Ru Hee, Dayna Cheng, Yu-Hong Chen, Sheng-Hsuan Wang, Chiao-Hsuan Chao, Sheng-Wen Huang, Pin Ling, Shu-Wen Wan, Chih-Peng Chang, Justin Jang Hann Chu, Trai-Ming Yeh, Jen-Ren Wang

**Affiliations:** 1 Department of Medical Laboratory Science and Biotechnology, College of Medicine, National Cheng Kung University, No.1, University Road, Tainan, 701 Taiwan; 2 Institute of Basic Medical Sciences, College of Medicine, National Cheng Kung University, Tainan, Taiwan; 3 Department of Medical Laboratory and Regenerative Medicine, MacKay Medical College, New Taipei, Taiwan; 4 National Mosquito-Borne Diseases Control Research Center, National Health Research Institutes, Tainan, Taiwan; 5 Department of Microbiology and Immunology, College of Medicine, National Cheng Kung University, Tainan, Taiwan; 6 Center of Infectious Disease and Signaling Research, National Cheng Kung University, Tainan, Taiwan; 7 Department of Microbiology and Immunology, Infectious Diseases Translational Research Program, Yong Loo Lin School of Medicine, National University of Singapore, Singapore, Singapore; 8 National Institute of Infectious Diseases and Vaccinology, National Health Research Institutes, Tainan, Taiwan

**Keywords:** Dengue virus, Non-structural protein 1, Type I interferon, Pro-inflammatory cytokines

## Abstract

**Background:**

The largest dengue virus 2 (DENV2) outbreak occurred in Taiwan in 2015, resulting in many fatalities. We therefore aim to identify crucial genetic variations which determine the virulence of the 2015 Taiwan outbreak strains.

**Methods:**

We compared the 2015 Taiwan DENV2 sequences to the pre-2015 sequences. Reverse genetics (rg) viruses with substitutions were produced and the viral growth kinetics were investigated. We treated A549 cells with interferon (IFN) to determine the interferon-stimulated genes (ISGs) expression and STAT1 phosphorylation in the rg viral infection and plasmid transfection systems. IFN and pro-inflammatory cytokines levels were measured upon DENV infection using ELISA.

**Results:**

The rgNS1-K272R mutant showed faster replication in IFN-I producing cells compared to wildtype (WT) virus. Results revealed that NS1-K272R substitution contributed to higher soluble NS1 secretion and evade the antiviral response by suppressing the expression of ISGs and STAT1 phosphorylation compared to NS1-WT. Infection with rgNS1-K272R induced higher secretion of pro-inflammatory cytokines through the activation of canonical nuclear factor-kappa B (NF-κB) signaling pathway.

**Conclusions:**

Our results revealed that the DENV NS1 amino acid substitution affects the NS1 ability in immune evasion, which may contribute to the largest dengue outbreak in Taiwan since the 1990s.

**Supplementary Information:**

The online version contains supplementary material available at 10.1186/s12929-024-01116-4.

## Background

Dengue virus (DENV) is an arbovirus which affects millions of people annually and is mainly transmitted by the *Aedes* species mosquitoes in tropical and sub-tropical regions. DENV infections usually cause mild, self-limiting diseases or even no symptoms, however, dengue can also cause life-threatening conditions such as dengue hemorrhagic fever (DHF) and dengue shock syndrome (DSS) when exposed to young infants, the geriatric population, and patients with comorbidities [1, 2]. Despite the emergence and re-emergence of dengue, it is now a global public health burden and there is still no specific antiviral drug against DENV infections.

DENV is a positive sense, single-stranded RNA virus. The genome of DENV is encoded into a single polyprotein and is then cleaved into three structural proteins consisting of the envelope (E), pre-membrane (M), and capsid (C) proteins, and seven non-structural proteins including non-structural protein 1 (NS1), NS2A, NS2B, NS3, NS4A, NS4B, and NS5 proteins. DENV NS1 is a glycoprotein which can be secreted from infected cells and is found circulating in the blood of dengue patients. The elevated levels of circulating NS1 correlates with DHF which can be used as a diagnostic biomarker monitoring the severity of dengue disease in the acute phase of infection [3, 4]. Due to the hydrophobic properties and membrane affinity of NS1, the NS1 localizes at the vesicle packet site which is an endoplasmic reticulum (ER)-derived ultrastructure formed by the invagination of the ER membrane containing the viral non-structural proteins as well as the double-stranded RNA. Therefore, NS1 may not contribute to RNA replication directly but acts as a scaffolding protein leading to the formation of vesicle packets [5, 6]. It has also been reported that the DENV NS1 activates dendritic cells and peripheral bone marrow macrophages producing pro-inflammatory cytokines and chemokines such as tumor necrosis factor alpha (TNF-α), interleukin 6 (IL-6), IL-8, and IL-1β which trigger the disruption of intercellular junctions [7–10]. Dysregulation of cytokine production has been proposed to be the mechanism of vascular leakage in DHF and DSS, therefore, the elevated levels of these pro-inflammatory cytokines are important predictors of the disease severity for dengue patients [10–13]. In addition to vascular leakage, DENV NS1 is also involved in immune evasion as it blocks several complements including C4, C4b, and C9 [14–16].

RNA viruses tend to have a high mutation rate due to the low fidelity of their RNA-dependent RNA polymerase (RdRp). The average rate of RNA viruses is one mutation per genome per cycle [17]. In previous studies, an amino acid substitution, T164S, in the NS1 region was found to be associated with the 1997 Cuban DENV2 outbreak [18, 19]. Furthermore, the reverse genetics (rg) virus containing the T164S amino acid substitution increased the secreted NS1 (sNS1) secretion in mammalian cells and human peripheral blood mononuclear cells (PBMCs). The sNS1 purified from T164S mutant virus was also used to treat the PBMCs, showing higher pro-inflammatory cytokines production than wildtype (WT) sNS1 [20]. A single amino acid substitution, NS1-G53D, was identified in the DENV2 16681 strain which affects the infection and dissemination of virus in both mosquito and mammalian cells [21]. Scaturro et al. found that alanine substitutions at NS1 residues S114, W115, D180, and T301 altered the production of infectious particles, however, the mutants showed minor effects on viral RNA replication [22]. Besides, the NS1 sequence of the DENV2 Cosmopolitan genotype virus may contribute to the higher viral titers in the blood and mortality in interferon-receptor knock-out mice compared to the Asian strain [23].

Type I interferon (IFN-I) response is the front line of host defense against viral infections. During DENV infection, the viral nucleic acids and other pathogen associated molecular proteins (PAMPs) are recognized by pattern recognition receptors (PRRs) such as toll-like receptors (TLRs) and retinoic acid inducible gene I (RIG-I), leading to IFN-I production including IFN-α and IFN-β. Once IFN-I binds to its receptor, the canonical signal transducer and activator of transcription 1 (STAT1)–STAT2–IFN-regulatory factor 9 (IRF9) signaling complex activates the IFN-stimulated response elements (ISREs) in gene promoters, which induces the expression of a large number of IFN-stimulated genes (ISGs). In previous studies, the NS proteins of flaviviruses has been shown to be associated with the inhibition of IFN-I response. For instance, the DENV NS2B/3 complex suppresses IFN-I production by cleaving the stimulator of interferon genes (STING) in human monocyte derived dendritic cells [24]. Additionally, the NS proteins of DENV hijack the IFN-I signaling pathway by targeting adaptor mitochondrial antiviral-signaling (MAVS) protein or degrading STAT2 protein [25, 26]. NS1 of ZIKV hampers the induction of IFN-I to evade the IFN-mediated antiviral response [27]. Furthermore, NS1 of WNV suppresses the production of IFN-β by targeting RIG-I and melanoma differentiation-associated gene 5 (MDA5) [28].

In 2015, there was a severe dengue outbreak which occurred in Southern Taiwan and over 40,000 cases were reported, including 228 deaths [29]. In this study, we identified an amino acid substitution, K272R, in the NS1 region from the 2015 Taiwan DENV2 outbreak strains. We found that the NS1-K272R substitution contribute to immune evasion by suppressing the phosphorylation of STAT1 protein and the downstream expression of ISGs. The results also suggest that the K272R mutant secretes higher levels of soluble NS1 which further induced the activation of canonical nuclear factor kappa B (NFκB) signaling pathway and the production of pro-inflammatory cytokines compared to WT virus.

## Methods

### Cell culture

All cell lines were obtained from ATCC and maintained at 37 ℃ with 5% CO_2_. Baby hamster kidney (BHK-21) cells (ATCC: CRL-12071) and A549 cells (ATCC: CCL-185) were cultured in Dulbecco’s Modified Eagle Medium (DMEM; Gibco) supplemented with 10% fetal bovine serum (FBS; Hyclone) and 2% penicillin/streptomycin (P/S; Gibco). Vero cells (ATCC: CCL-81) were cultured in Eagle’s Minimum Essential Medium (EMEM; Gibco) supplemented with 10% FBS, 2% P/S, and 1% sodium pyruvate (Gibco), and 293 T cells (ATCC: CRL-3216) were cultured in DMEM supplemented with 10% FBS and 1% sodium pyruvate. For M1 macrophage differentiation, THP-1 cells (ATCC: TIB-202) were first cultured in RPMI medium supplemented with 10% FBS. To induce macrophage differentiation, THP-1 cells (2 × 10^5^/mL) were seeded in 48-well plates and treated with PMA (100 nM) for 20–24 h, followed by a 48h resting period. The cells were then incubated in RPMI medium containing 10% FBS, IFN-γ (20 ng/mL), and LPS (100 ng/mL) for an additional 24 h to achieve M1 polarization.

### Phylogenetic analysis

All DENV2 NS1 sequences of Asian countries including China, India, Indonesia, Singapore, Malaysia, Thailand, Vietnam, and the Philippines from 1995 to 2019 available on GenBank were downloaded (accessed on March 1, 2024). Two different sequence datasets were generated for phylogenetic analysis. The first dataset was composed of all Taiwan DENV2 NS1 sequences in GenBank from 1995 to 2014 including 22 NS1 sequences of 2015 Taiwan outbreak strains as well as 17 global reference strains (accession nos. KU509270, MF004385, AY037116, KT87555, JF327392, DQ448231, GU131924, U87411, NC001474, HQ026763, AB122022, KM587709, M29095, AY702040, AF100469, MK564477, and EF105384). We randomly selected representative strains from DENV2 isolates of Asian countries into another dataset, 22 of the 2015 Taiwan outbreak strains, and 17 global reference strains were also included. Phylogenetic tree was inferred from the alignment using the maximum likelihood approach generated in IQ-TREE software version 2.3.3 with bootstrapping by 1000 replicates and visualized using FigTree 1.3.1 programs.

### Genetic variation analysis

We previously sequenced 22 DENV2 isolates (accession nos, OR593362-OR593383) from the 2015 outbreak in Taiwan using next-generation sequencing (NGS) [30]. The consensus sequences of each isolate were compared to 29 Taiwan DENV2 strains before 2015 in GenBank to determine the amino acid substitutions of this outbreak.

### Construction of infectious cDNA clones

The DENV2 (16681 strain, Accession no.: KU725663.1) was used as template for infectious clone construction. The construction of infectious clones was similar as previously described [30]. In brief, the plasmid pDL-DENV2-EGFP-C60-10062016-A2 was used as template to perform site-directed mutagenesis producing reverse genetics (rg) viruses containing each amino acid substitution isolated from the outbreak. The specific primers used in site-directed mutagenesis were listed in Table S1 The plasmids were transfected into BHK-21 cells (8 × 10^5^/well) in 6-well plates using PolyJet™ in vitro DNA Transfection Reagent (SignaGen) according to manufacturer’s instructions. The rg viruses were harvested at 7–9 days post-transfection when the fluorescence was observed on more than 75% of cells. Then, the rg viruses were passaged twice in Vero cells and collected for further experiments.

### Construction of recombinant NS1 clones

The full-length DENV2 NS1 sequence was cloned into pGEM-T vector by TA cloning using the plasmids of WT virus and K272R mutant as template for the amplification of NS1 sequence. The sequences of recombinant NS1 clones were then constructed into pTT5-glu with Kozak consensus sequence in front of the beginning of NS1 sequence which is important for efficient translation initiation. The primers used in the construction of recombinant NS1 clones were listed in Table S1.

### Infection and transfection

DMEM (2% FBS, 2% P/S) and EMEM (2% FBS, 2% P/S, 1% sodium pyruvate) viral medium were used in infection experiments to analyze the growth kinetics, viral fitness and stability, ISGs mRNA expression as well as cytokine secretion. A549 cells (6 × 10^5^/well) were seeded in 6-well plates and incubated for 24 h and differentiated M1 macrophage (~ 1 × 10^5^/well) were well differentiated in 48-well plates first. The cells were then infected by rg viruses with a 1 h adsorption period. For the transfection of pTT5-NS1 plasmids, 293 T cells (8 × 10^5^/well) were seeded in 6-well plates for 24 h. The plasmids were transfected into cells using HyFect® Transfection Reagent (Leadgene Biomedical, Taiwan) following manufacturer’s instruction and the old medium was replaced by the FreeStyle™ 293 Expression Medium (ThermoScientific) after 24 h incubation.

### IFN-α treatment

The infected or transfected cells were treated with human IFN-Alpha 2b (2000 IU/mL) for 6 h at 3 days post-infection before the sample was collected for quantitative PCR of ISGs including *IFIT1, ISG15,* and *MxA*. For the inhibition assay of STAT1 phosphorylation, the infected or transfected cells were treated with human IFN-Alpha 2b (2000 IU/mL) for 1 h before the sample was collected for Western blot analysis.

### ELISpot assay for rgDENV quantification

Vero cells (2 × 10^4^/well) were seeded in 96-well plates for 24 h and infected with 30 µL of ½ log serially diluted rg viruses. After 1 h incubation, 150 µL of overlay medium, which contains EMEM viral medium and 1% methyl cellulose, was added into each well. The supernatant was aspirated at 4 days post-infection and the cells were washed with PBS twice. The cells were fixed with 80% methanol and freeze-thawed before immunostaining. The methanol was removed, and the cells were blocked with 5% skimmed milk in PBS. After 30 min of blocking at room temperature, the cells were stained with primary antibodies, mouse anti-dengue complex monoclonal antibody (MAB8705, Millipore) at 1:2000 dilution and incubated at 37 °C for 2 h. Then, the cells were washed 4 times with PBS-T (0.05% Tween 20 in PBS) before staining with goat anti-mouse IgG conjugate HRP (KPL (#5220-0460), LGC Seracare). After 2 h incubation, the cells were washed 6 times with PBS-T and the TrueBlue Peroxidase Substrate (KPL (#5510-0030); LGC Seracare, Milford, MA, United States of America) was added to each well and incubated for 30 min in the dark. The photographs of each well were then taken using the ImmunoSpot system and the focus forming units per milliliter (FFU/mL) was then calculated.

### Growth kinetics

A549 cells and Vero cells (2 × 10^5^/well) were seeded in 24-well plates for 24 h and infected by rg viruses at MOI 0.01. The viruses were collected at 0-, 1-, 2-, 3-, and 4-days post-infection then titrated by ELISpot assay.

### Viral competition assay

Vero cells (1 × 10^5^/well) were seeded in 24-well plates for 24 h and infected by WT virus, mutant viruses, or 1:1 mixture of WT and each mutant. After each passage, 100 µL each group of viruses were used for the following passage up to P5. Sanger sequencing was then performed to determine the major variant at P1 and P5.

### NS1 ELISA

The procedure of this experiment was similar to the commercialized ELISA kits except that the capture antibodies and detection antibodies were in-house products while the standard was purchased additionally. The capture antibody was a 1:1 mixture of 31B2 and 33D2 anti-NS1 monoclonal antibodies, and the detection antibody was biotin-labelled 33D2 anti-NS1 antibody. The working concentration of each antibody were 5 µg/mL and 2 µg/mL, respectively. The standard used in this experiment was purchased from the Native Antigen Company, which is DENV2 NS1 hexamer produced in mammalian HEK293 cells with purity higher than 95% determined by sodium dodecyl sulphate–polyacrylamide gel electrophoresis (SDS-PAGE).

### Quantitative real-time PCR analysis

The infected or transfected cells were treated with human IFN-alpha 2b (Alpha 2) Protein (PBL Assay Science) before the samples were collected for further analysis. The mRNA of infected A549 cells and M1 macrophages were extracted using the Genomic Total RNA Extraction Kit (RBC Bioscience) and then 1 µg of total RNA was reverse transcribed into cDNA using MMLV Reverse Transcriptase (Promega) with random primers. Quantitative real-time PCR (qPCR) was performed with 2 × qPCRBIO SyGreen Blue Mix in StepOnePlus Real-Time PCR System. The primers of IFN-β, ISGs, and pro-inflammatory cytokines used in the qPCR were listed in Table S2.

### Enzyme linked immunosorbent assay (ELISA)

The culture supernatants from infected A549 cells and stimulated M1 macrophages were collected to measure cytokine levels. For A549 cells, IFN-I and pro-inflammatory cytokines, including IFN-β, IL-6, and IL-8, were quantified. For M1 macrophages, IL-6 and IL-8 levels were measured. All cytokine measurements were performed using Human DuoSet ELISAs (Bio-Techne) according to the manufacturer’s instructions.

### Western blot analysis

The cell lysates were collected, and the total protein levels were quantitated by Bradford assay (BioRad). The pSTAT1 (9172, Cell Signaling Technology), STAT1 (9167, Cell Signaling Technology), pp65 (6956, Cell Signaling Technology), p65 (3303, Cell Signaling Technology), NS1 (LDG0001YA, Leadgene Biomedical), and internal control GAPDH (GTX627408, Genetex) were captured respectively by primary antibodies followed by HRP-conjugated anti-mouse (474-1082, Millipore) or anti-rabbit (7074, Cell Signaling Technology) antibodies. The bound secondary antibodies were then detected by WesternBright ECL HRP substrate (Advansta). The results were quantified using the ImageJ software.

### Statistical analysis

All statistical analyses were performed using one-way analysis of variance (ANOVA) or two-way ANOVA in GraphPad Prism 7 software. A value of p < 0.05 represents statistical significance.

## Results

### Identification of amino acid substitutions in Taiwan 2015 DENV2 outbreak

**Fig. 1:**
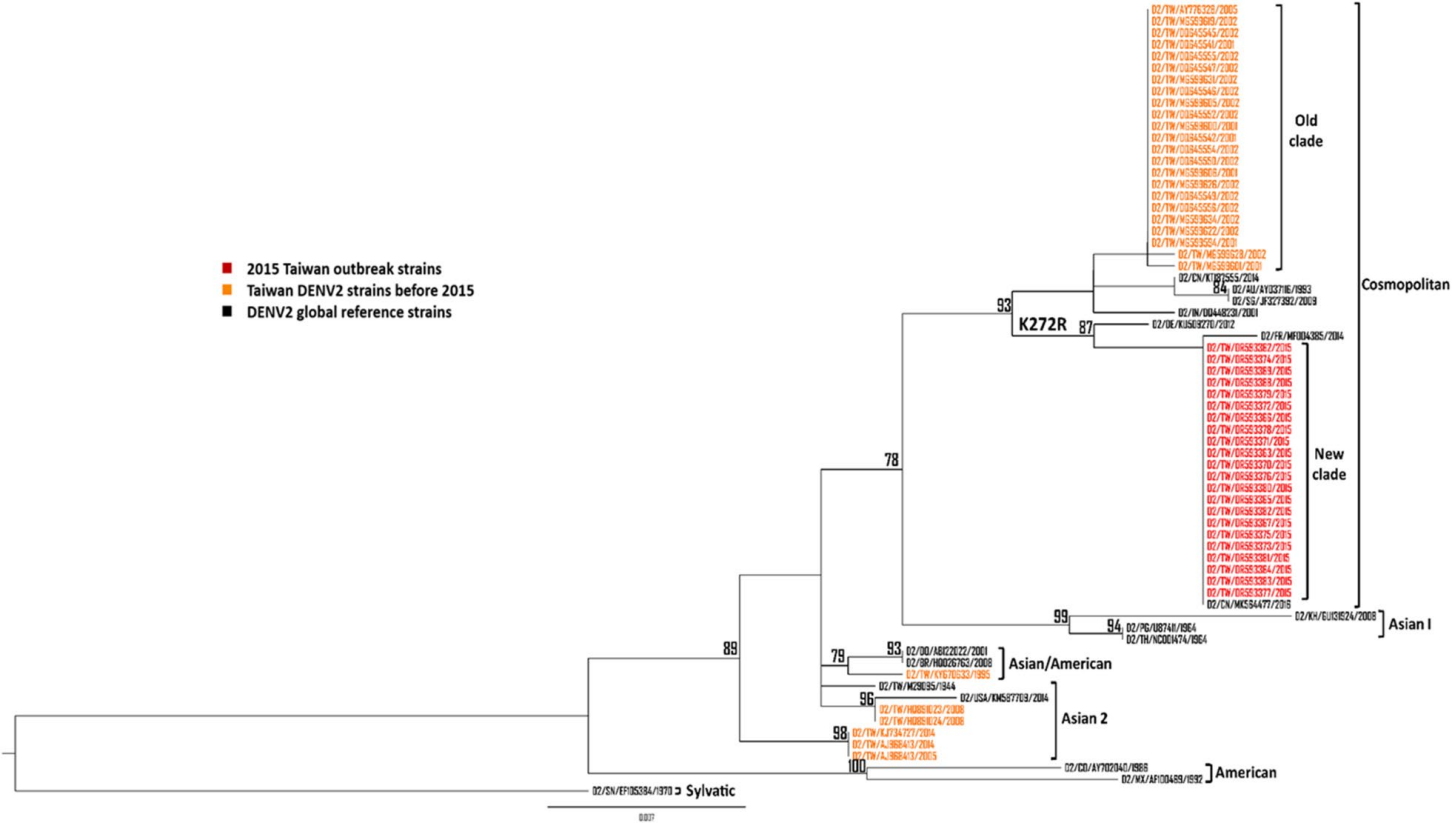
Phylogenetic tree of Taiwan DENV2 strains from 1995 to 2015 based on the DENV2 NS1 full-length sequence. The maximum likelihood phylogenetic tree was constructed using the IQ-TREE program with 1000 bootstrap replications. The sequences of DENV2 Taiwan strains from 1995 to 2014 were collected from GenBank while the 2015 DENV2 outbreak strains were previously sequenced with the Illumina Miseq platform [30]. The 2015 Taiwan outbreak strains were marked in red, the Taiwan DENV2 strains from 1995 to 2014 were marked in orange, and the global reference strains were marked in black. Virus names were shown as country, accession number, and reported year of each sequence. Numbers on nodes were bootstrap support value exceeding 75%

**Fig. 2:**
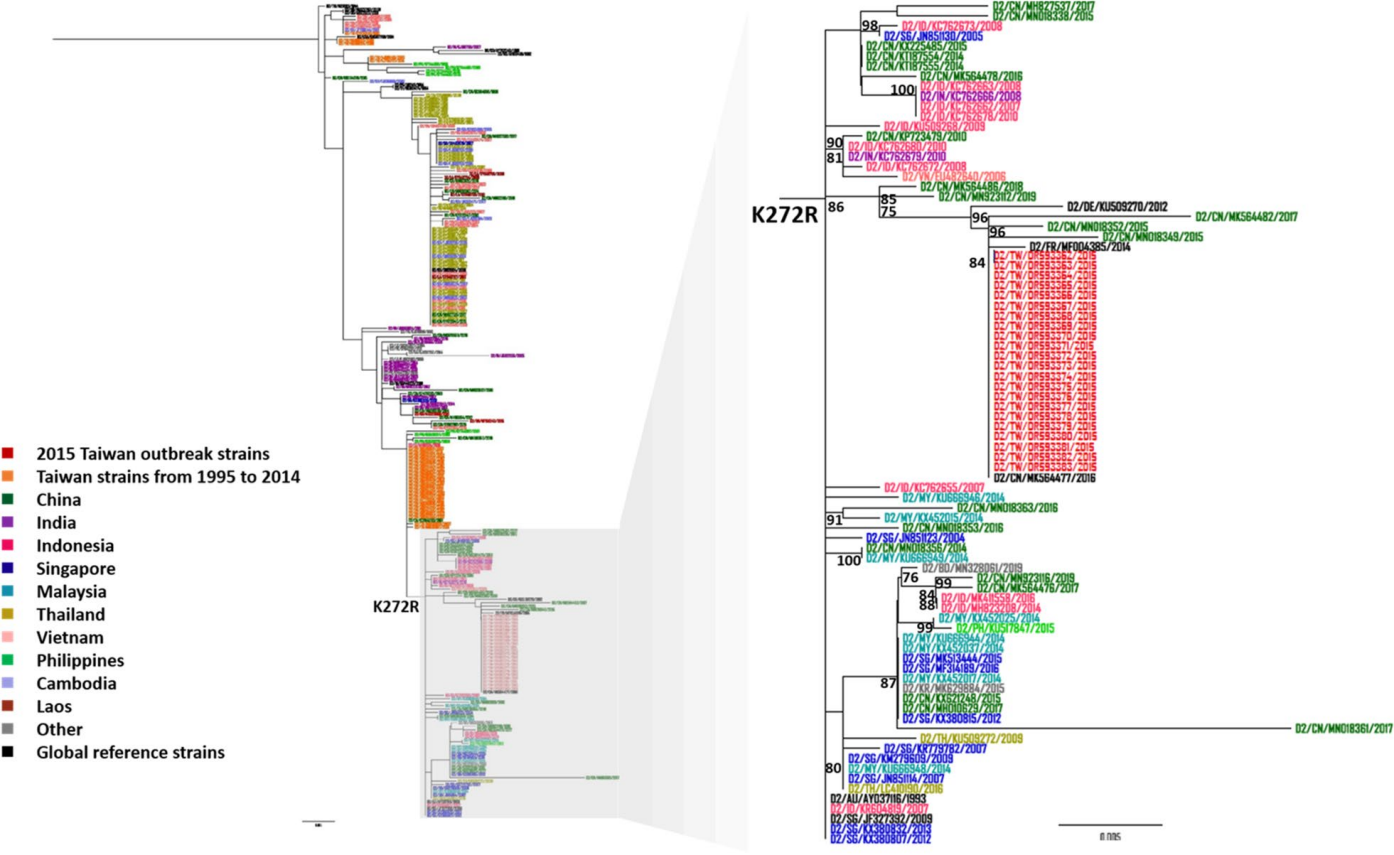
Phylogenetic tree of DENV2 strains of Asian countries from 1995 to 2019 based on the DENV2 NS1 full-length sequence. The maximum likelihood phylogenetic tree was constructed using the IQ-TREE program with 1000 bootstrap replications. The sequences of DENV2 of Asian countries from 1995 to 2019 were collected from GenBank while the 2015 DENV2 outbreak strains were previously sequenced with the Illumina Miseq platform [30]. The 2015 Taiwan outbreak strains were marked in red, the global reference strains were marked in black, and the DENV2 strains of Asian countries from the other countries were marked in different colors. Virus names were shown as country, accession number, and reported year of each sequence. Numbers on nodes were bootstrap support value exceeding 75%. TW: Taiwan; CN: China; IN: India; ID: Indonesia; SG: Singapore; MY: Malaysia; TH: Thailand; VN: Vietnam; PH: Philippines; KH: Cambodia; LA: Laos

To investigate the amino acid substitutions which may contribute to the 2015 Taiwan dengue outbreak, we collected the sequences of Taiwan DENV2 in GenBank from 1995 to 2014 and compared them to the sequences of 22 strains clinical isolates from the 2015 dengue outbreak. A total of 11 amino acid substitutions were identified in the capsid protein (K73R), NS1 (P73Q, H224N, K272R, D278E), NS2A (S39T, T104A, K166R, I171T), NS3 (V395I), and NS5 (K387R). Notably, all the viruses isolated by inoculation of the patient serum onto the mosquito cells, C6/36 cells, consistently possessed the 11 amino acid substitutions mentioned above (Table S3). The 2015 Taiwan outbreak strains were previously classified as DENV2 Cosmopolitan genotype [31]. As previously mentioned, NS1 plays an essential role in viral replication, immune evasion, and immunopathogenesis. To understand the evolutionary relationship of DENV2 in Taiwan from 1995 to 2015 based on the NS1 full-length sequence, we performed phylogenetic analysis via the IQ-TREE software. The result showed that the NS1 sequence of the 2015 outbreak strains were highly similar to the global DENV2 Cosmopolitan genotype reference strains. Furthermore, the Cosmopolitan genotype strains were split into two branches. The NS1 of 2015 Taiwan outbreak strains revealed a new clade, indicating that a clade replacement had occurred (Fig. 1). We further collected the sequences of DENV2 strains from Asian countries including China, India, Indonesia, Singapore, Malaysia, Thailand, Vietnam, and the Philippines from 1995 to 2019 to perform phylogenetic analysis based on the NS1 full-length sequence. The 2015 outbreak strains were closely related to strains circulating in China from 2015 to 2017 (Fig. 2). Interestingly, we found that the strains isolated from the other Asian countries which belonged to the same clade as the 2015 outbreak strains only shared R272 amino acid among the four amino acid substitutions in the NS1 region we mentioned above (Table S4).

### K272R mutant virus replicated faster than WT virus in type I IFN producing cells

**Fig. 3:**
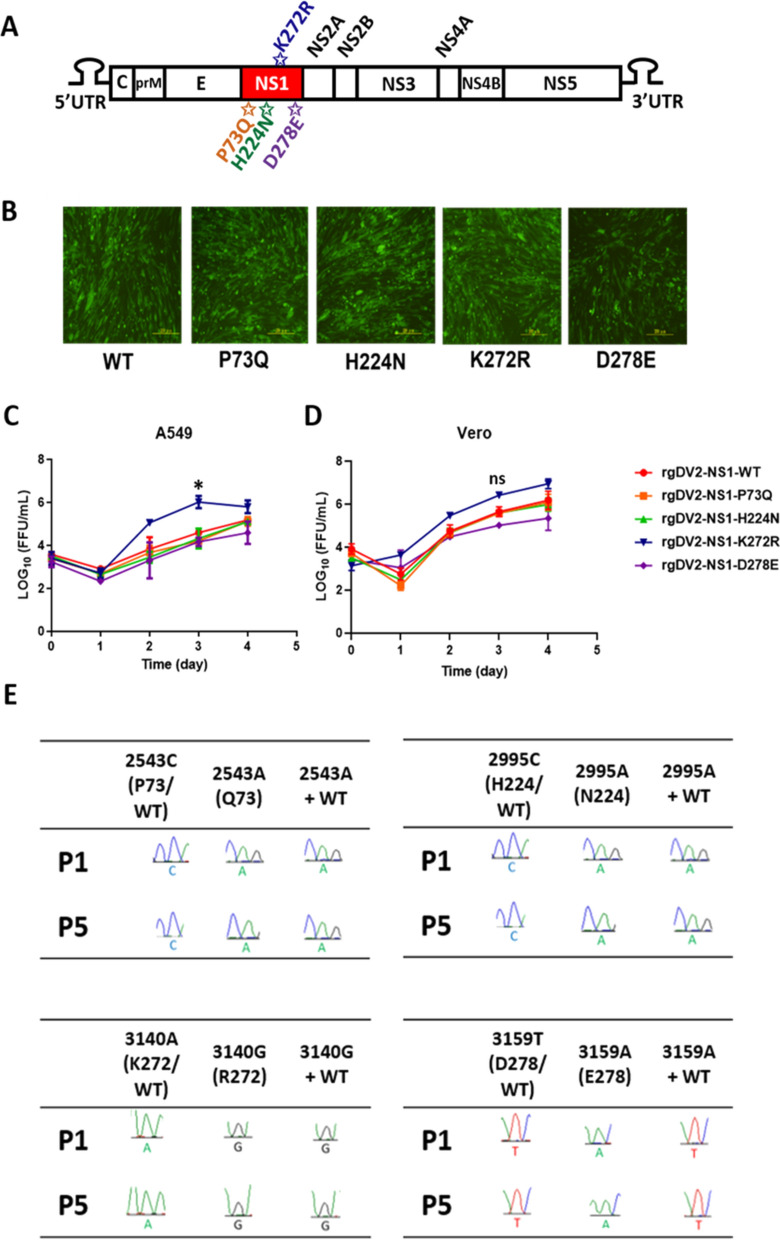
K272R mutant virus replicated faster than WT virus in type I IFN producing cells. **A** Schematic diagram of NS1 amino acid mutations of Taiwan 2015 DENV2 outbreak strains. The red region represents the NS1 protein of DENV2. The stars indicate the amino acid substitutions isolated from the 2015 Taiwan dengue outbreak. **B** Production of DENV2-EGFP rg viruses containing each amino acid substitution in BHK-21 cells. The fluorescent images were captured under 10 × magnification of fluorescent microscope. **C**, **D** Growth kinetics of each rg virus in **C** A549 cells and **D** Vero cells. **E** Viral competition assay of each rg virus in Vero cells. Vero cells were infected with WT virus, mutant viruses or 1:1 mixture of WT and each mutant, then each group of viruses were passaged up to P5. Sanger sequencing was performed to determine the dominant variant at P1 and P5. All data are representative data from at least two independent experiments with comparable results and plotted as mean ± SEM with *p < 0.05 and ns, p > 0.05 by two-way ANOVA. WT: wildtype

As is known, NS1 plays an essential role in viral replication [32]. Therefore, using site-directed mutagenesis, we produced DENV2-EGFP rg viruses that contain each amino acid substitution in the NS1 region of the 2015 Taiwan outbreak strain including P73Q, H224N, K272R, and D278E, to further investigate their viral growth kinetics (Fig. 3A, B). We first evaluated the replication kinetics of each rg virus in different mammalian cell lines including A549 cells and Vero cells. The cell lines were infected with each rg virus at a low multiplicity of infection (MOI) of 0.01. The virus samples were titrated by ELISpot assay to obtain the viral titers at 0-, 24-, 48-, 72-, and 96-h post-infection (hpi). The replication of NS1-K272R mutant virus reached a peak at 72 hpi and declined at 96 hpi in A549 cells, while the viral titers of WT and the other mutants were lower than K272R mutant consistently, indicating that the K272R mutant replicated faster than WT and the other mutants in A549 cells (Fig. 3C). However, there was no significant difference in the growth kinetics of each rg virus in Vero cells, which is an IFN-I deficient cell line (Fig. 3D). We found that the rapid replication of rgDENV2-NS1-K272R was only seen in the A549 cells, which may indicate that IFN-I plays a certain role in viral growth inhibition. Hence, we selected Vero cells for the viral competition assay in an aim to exclude the effect of IFN-I inhibition. A viral competition assay was then carried out to investigate the fitness and stability of each amino acid substitution. Each mutant virus was mixed with the WT virus in a ratio of 1:1 and passaged up to five passages (P5). The sequencing results showed that except for the D278E mutant, the other mutant viruses successfully outcompeted the WT virus, showing competitive survival advantage (Fig. 3E).

### K272R mutant promoted higher secretion of soluble NS1 (sNS1)

**Fig. 4:**
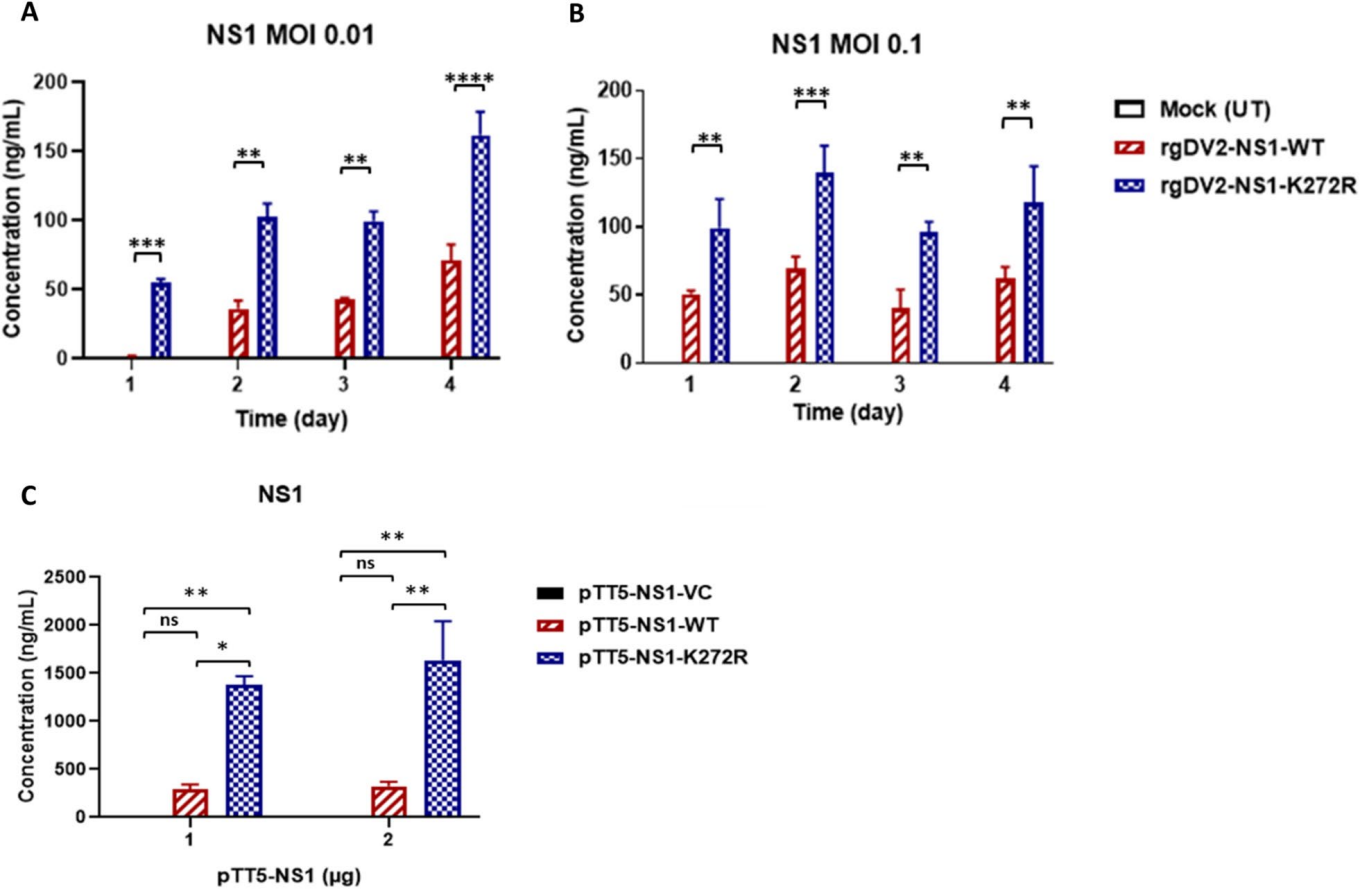
K272R mutant promoted higher secretion of soluble NS1. **A**, **B** A549 cells infected with K272R mutant or WT virus at or MOI 0.01 or MOI 0.1 for the indicated time points. **C** 293 T cells were transfected with 1 µg or 2 µg of plasmid overexpressing NS1-K272R or NS1-WT. **A**–**C** NS1 concentration in culture supernatants were measured by ELISA. All data are representative data from at least two independent experiments with ****p < 0.0001, ***p < 0.001, **p < 0.01, *p < 0.05 and ns, p > 0.05 by two-way ANOVA

According to previous studies, NS1 substitution influences the secretion of sNS1 in Huh7 cells [33]. We therefore attempted to determine the sNS1 level after rg virus infection. A549 cells were infected by rgDV2-NS1-K272R and rgDV2-NS1-WT at MOI 0.01 and MOI 0.1 and the sNS1 expression level in the culture supernatants was measured. The concentrations of NS1 secreted from rgDV2-NS1-K272R-infected cells were consistently higher than rgDV2-NS1-WT at different time points (Fig. 4A, B). Furthermore, we transfected pTT5-NS1 plasmids overexpressing NSI-K272R and NS1-WT, respectively in 293 T cells, then measured the NS1 in the culture supernatants using NS1 ELISA. The results showed that the cells transfected with pTT5-NS1-K272R secreted more NS1 compared to cells overexpressing NS1-WT, indicating that the K272R amino acid substitution increased the secretion of sNS1 (Fig. 4C).

### Infection of K272R mutant virus showed lower ISGs expression compared to WT virus

**Fig. 5:**
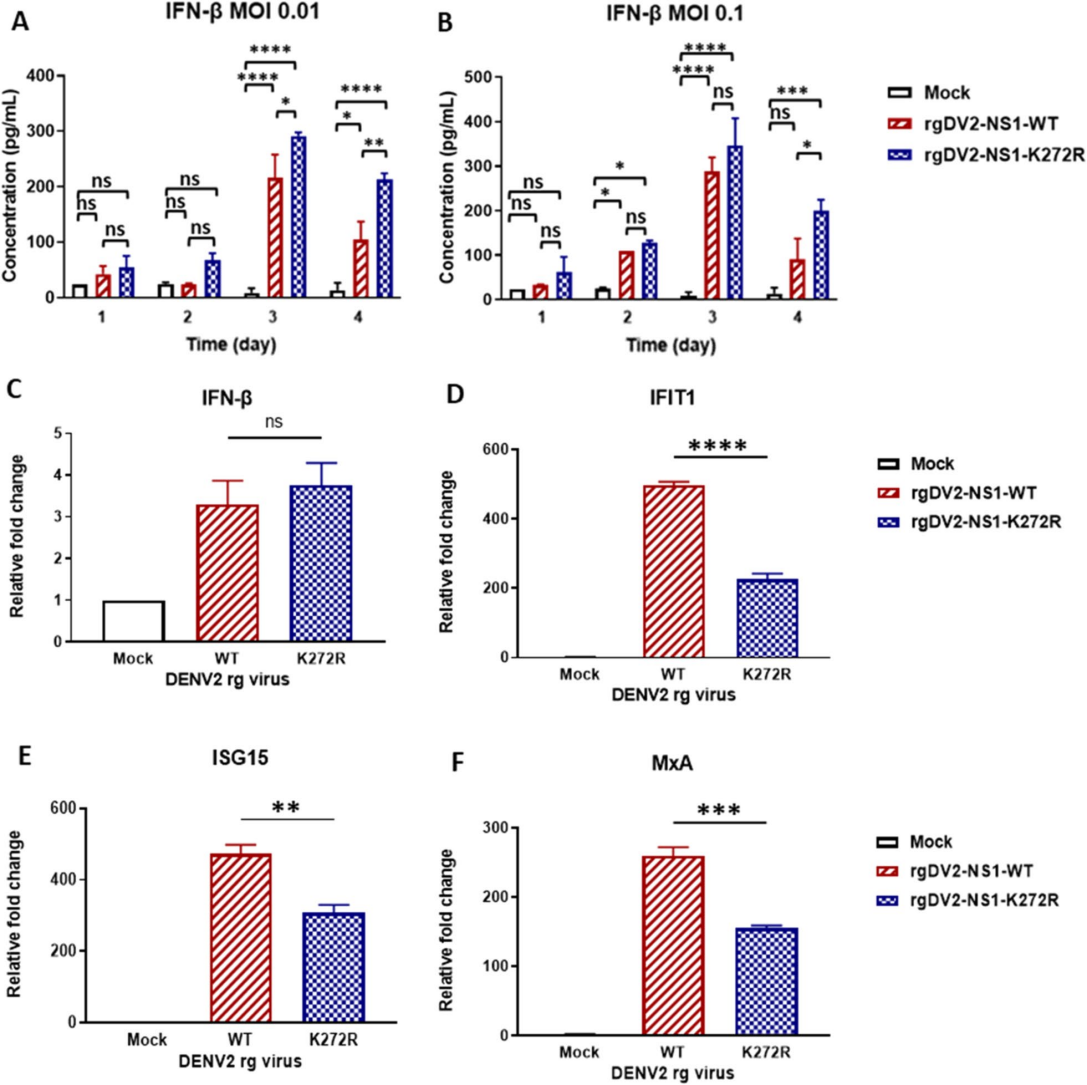
Infection with K272R mutant virus showed lower ISGs expression compared to WT virus. **A**, **B** A549 cells were infected with K272R mutant or WT virus at **A** MOI 0.01 or **B** MOI 0.1 for the indicated time points. IFN-β concentration in culture supernatants were measured by ELISA. **C**–**F** A549 cells were infected with K272R mutant or WT virus at MOI 0.01 for **C** 48 h or **D**–**F** 72 h, then the quantitative RT-PCR analysis of **C** *IFN-β* or ISGs **D** *IFIT1*, **E** *ISG15*, and **F** *MxA* were performed. All data are representative data from at least two independent experiments with ****p < 0.0001 and ns, p > 0.05 by **A**, **B** two-way ANOVA or **C**–**E** one-way ANOVA

Previous studies show that the NS of flaviviruses inhibits the IFN-I response through different mechanisms [27, 28]. Since the K272R mutant virus showed faster replication in A549 cells and induced higher levels of sNS1 secretion, we further investigated whether K272R mutant inhibits the IFN-I response by suppressing IFN-β secretion. The K272R mutant virus-infected cells secreted significantly higher concentrations of IFN-β than WT virus at 3- and 4-days post-infection (dpi), MOI 0.01 (Fig. 5A). The same phenomenon was also observed at 4 dpi, MOI 0.1 (Fig. 5B). Furthermore, the mRNA level of IFN-β induced by K272R mutant virus was found to be similar to WT virus at 2 dpi which was consistent with the ELISA results (Fig. 5C). These results suggested that the K272R mutant did not inhibit the IFN-I antiviral response by suppressing IFN-β production. However, compared to the WT virus, infection with the K272R mutant virus resulted in significantly lower expression of interferon-stimulated genes (ISGs), including *interferon-induced protein with tetratricopeptide repeats 1 (IFIT1)*,* interferon stimulated gene 15 (ISG15)*, and *Myxovirus resistance protein A (MxA)* (Fig. 5D–F), in a dose-dependent manner (Fig. S1). We also investigated the ISGs gene expression in the THP-1 derived M1 type macrophage after rgDENV2-NS1-K272R infection. The results revealed that significantly lower *IFIT1* and *MxA* expressions were detected compared to the WT virus infection (Fig. S2). Thus, we assumed that the K272R mutant may inhibit the IFN-I response by attenuating the expression of ISGs.

### K272R amino acid substitution contributed to immune evasion from IFN-I antiviral response

**Fig. 6:**
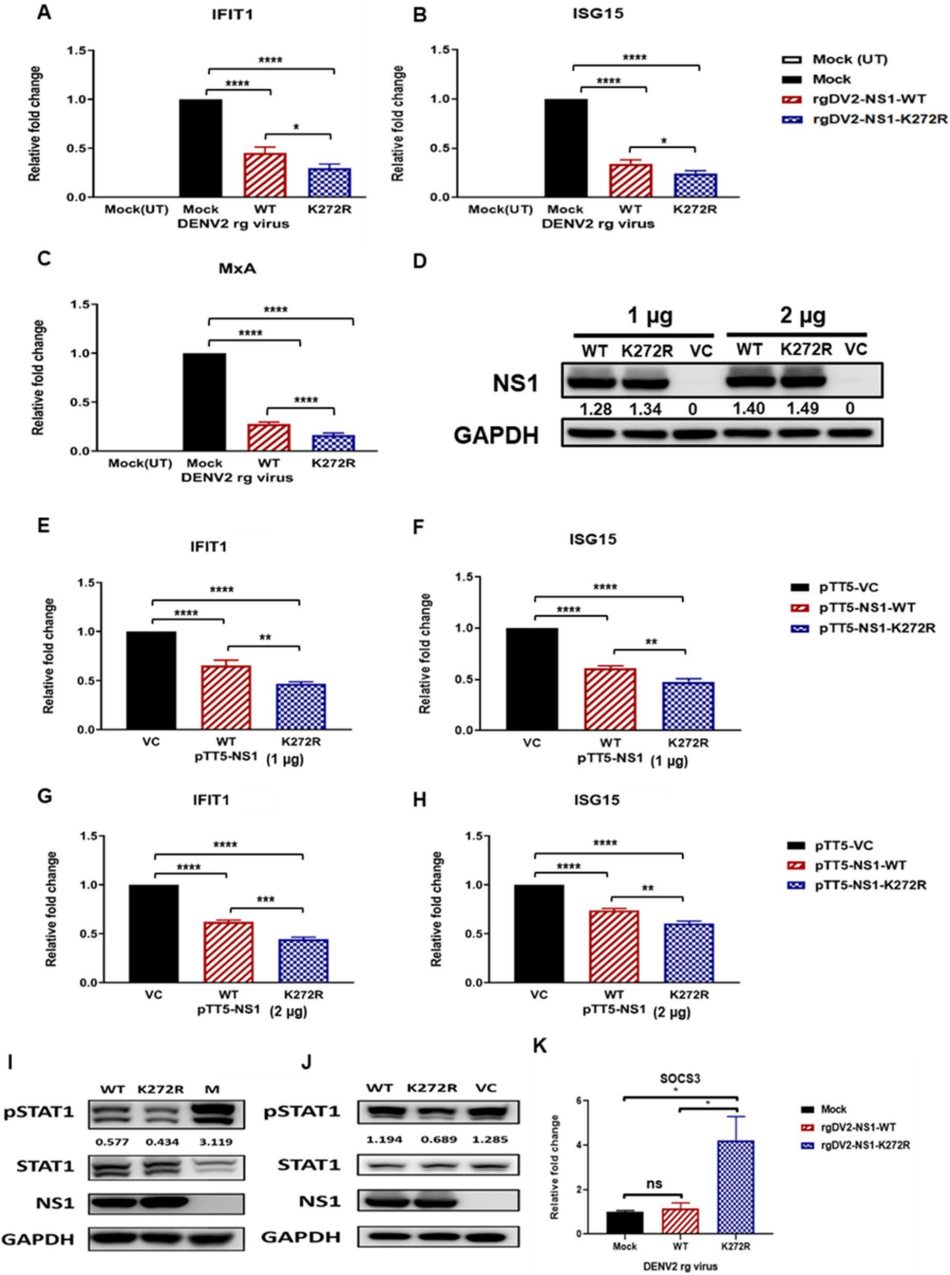
K272R amino acid substitution contributed to immune evasion from antiviral response. **A**–**C** A549 cells were infected with K272R mutant virus or WT virus at MOI 0.1 for 72 h followed by IFN-α treatment (1000 IU/mL) for 6 h. Quantitative RT-PCR analysis of ISGs (A) *IFIT1*, (B) *ISG15*, and **C** *MxA* were performed. **D** Immunoassay of extracts of 293 T cells transfected with 1 µg or 2 µg of plasmid overexpressing NS1-K272R or NS1-WT. (E–H) 293 T cells transfected with **E**, **F** 1 µg or **G**, **H** 2 µg of plasmids overexpressing NS1-K272R and NS1-WT, followed by IFN-α treatment (1000 IU/mL) for 6 h. Quantitative RT-PCR analysis of ISGs **E**, **G** *IFIT1* and **F**, **H** *ISG15* were performed. **I**, **J** STAT1 phosphorylation status was analyzed by western blot analysis. **I** A549 cells extracts with K272R mutant virus or WT virus infection at MOI 0.5 for 72 h and **J** 293 T cells were transfected with 2 µg of vector control, overexpressing NS1-WT and K272R mutant plasmids followed by IFN-α treatment (2000 IU/mL) for 1 h. The phosphorylated STAT1 protein expression was detected **K** A549 cells were infected with K272R mutant virus or WT virus at MOI 0.01 for 72 h and quantitative RT-PCR analysis of SOCS3 was performed. All data are representative data from at least two independent experiments with ****p < 0.0001, ***p < 0.001, **p < 0.01, *p < 0.05 by one-way ANOVA. UT: untreated; VC: vector control

To assess whether the higher viral titer of the K272R mutant virus contributes to immune evasion from the antiviral IFN-I response, we infected A549 cells with either the K272R mutant virus or the WT virus following IFN-α treatment, which upregulates the antiviral ISG response. We then evaluated the intracellular mRNA levels of ISGs to determine the impact. Infection with both K272R mutant virus and WT virus suppressed the expression of ISGs including *IFIT1*, *ISG15*, and *MxA*. As expected, infection with K272R mutant virus inhibited the mRNA abundance of ISGs more significantly than WT virus (Fig. 6A–C). Due to the higher viral titer of K272R mutant virus, there were more NS proteins expressed in the K272R mutant virus-infected cells. To confirm the role of NS1-K272R on ISGs inhibition, we transfected pTT5-NS1 plasmids into 293 T cells to express equivalent levels of NS1-K272R and NS1-WT intracellularly. Both the levels of intracellular NS1-K272R and NS1-WT were similar in 293 T cells (Fig. 6D). Upon IFN-α treatment, the expression of ISGs *IFIT1* and *ISG15* were suppressed in both NS1 expressed cells. Furthermore, NS1-K272R showed significantly higher inhibition of the ISGs expression when compared with NS1-WT at 1 µg and 2 µg plasmid transfection (Fig. 6E–H). To determine whether rgDV2-NS1-K272R hijacks the Janus kinase (JAK)-STAT signaling pathway, we first infected A549 cells with rgDV2-NS1-WT and rgDV2-NS1-K272R at MOI 0.5 and treated the cells with IFN-α for 1 h, then the phosphorylation of STAT1 protein was determined by Western blot analysis. As expected, both the rg viruses inhibited the phosphorylation of STAT1 protein compared to the mock-infected group. Furthermore, the rgDV2-NS1-K272R suppressed the expression of phosphorylated STAT1 (pSTAT1) protein more than rgDV2-NS1-WT (Fig. 6I). Similar phenomenon was also observed in the 293 T cells transfected with pTT5-NS1-WT and pTT5-NS1-K272R upon IFN-α treatment whereby the cells overexpressing NS1-K272R inhibited the phosphorylation of STAT1 protein more than the cells overexpressing NS1-WT (Fig. 6J). In addition, we also found that the rgDV2-NS1-K272R-infected cells showed higher mRNA expression of *suppressor of cytokine signaling 3 (SOCS3)* compared to the rgDV2-NS1-WT (Fig. 6K). Taken together, we suggest that NS1-K272R had a stronger ability to suppress the ISGs expression and STAT1 protein phosphorylation through the upregulation of *SOCS3* expression.

### K272R mutant virus promoted higher secretion of pro-inflammatory cytokines

**Fig. 7:**
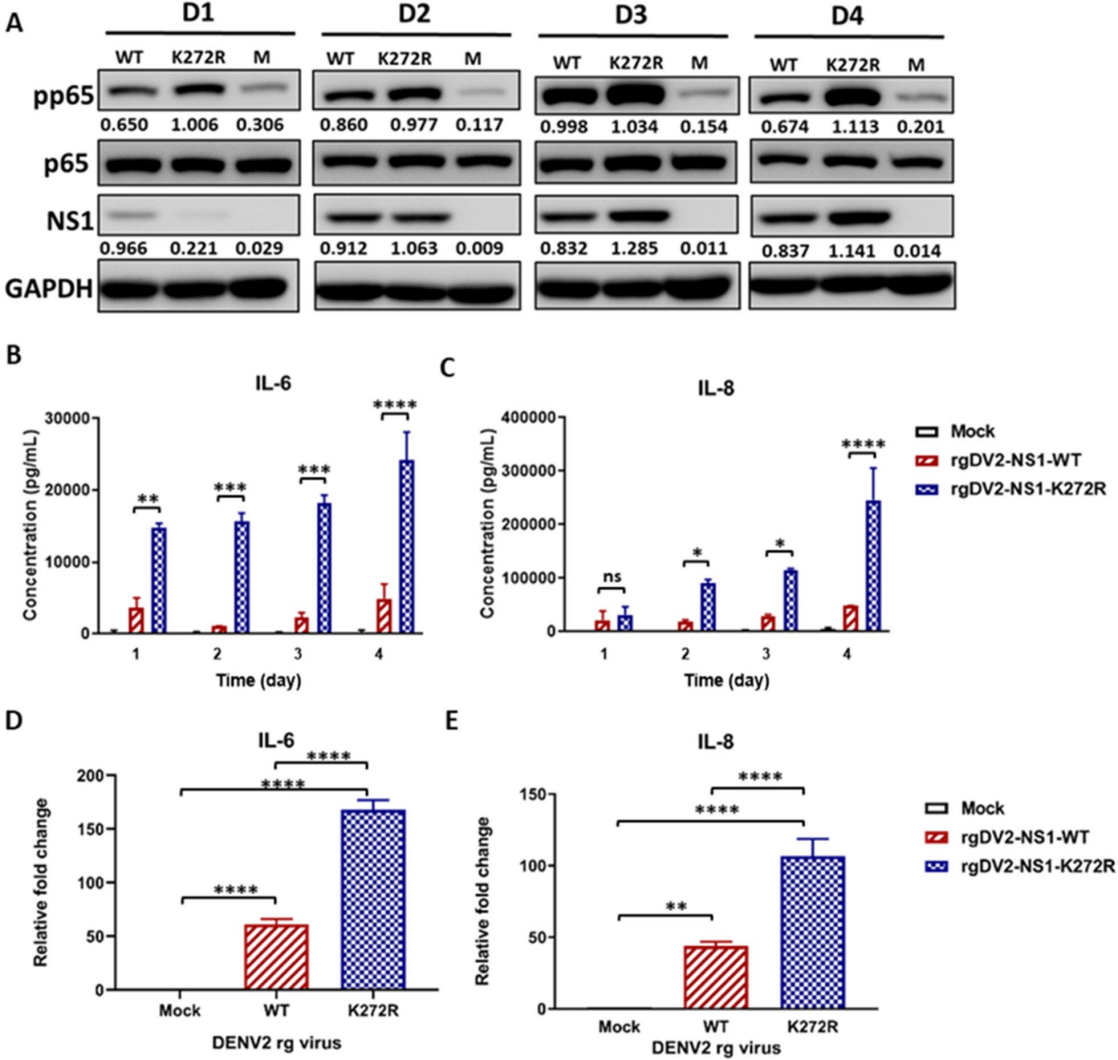
K272R mutant virus promoted higher secretion of pro-inflammatory cytokines. **A** Western blot of extracts of A549 cells infected with K272R mutant virus or WT virus at MOI 0.1 for 1–4 days (D1–D4). **B**, **C** A549 cells were infected with K272R mutant virus or WT virus at MOI 0.1 for the indicated time points. **B** IL-6 or **C** IL-8 concentration in culture supernatants were measured by ELISA. **D**, **E** A549 cells were infected with K272R mutant virus or WT virus for 48 h. Quantitative RT-PCR analysis of pro-inflammatory cytokines **D** IL-6 and **E** IL-8 were performed. All data are representative data from at least two independent experiments with ****p < 0.0001, ***p < 0.001, **p < 0.01 and *p < 0.05 by **B**, **C** two-way ANOVA or **D**, **E** one-way ANOVA

On the other hand, we considered whether rgDV2-NS1-K272R triggered a stronger activation of a pivotal mediator of inflammatory response, nuclear factor kappa B (NFκB), compared to rgDV2-NS1-WT. Thus, we infected A549 cells with rgDV2-NS1-WT and rgDV2-NS1-K272R at MOI 0.1 and the phosphorylation of p65 was detected by using the Western blot analysis. As shown in Fig. 7A, the phosphorylation of p65 increased during the DENV2 infection and reached a peak at 3 dpi. Furthermore, rgDV2-NS1-K272R triggered more phosphorylation of p65 than rgDV2-NS1-WT at different time points, indicating that rgDV2-NS1-K272R stimulated the activation of NF-κB more significantly than rgDV2-NS1-WT (Fig. 7A). We further determined whether rgDV2-NS1-K272R triggered a higher level of secretion of pro-inflammatory cytokines compared to rgDV2-NS1-WT. Thus, we infected A549 cells with rgDV2-NS1-WT and rgDV2-NS1-K272R at MOI 0.1, then the concentrations of pro-inflammatory cytokines IL-6 and IL-8 were measured by ELISA. The concentrations of IL-6 and IL-8 in culture supernatants were higher following rgDENV2-NS1-K272R infection compared to rgDENV2-NS1-WT at various time points (Fig. 7B, C) in a time and dose-dependent manner (Fig. S3). Additionally, the mRNA expression level of *IL-6* and *IL-8* were also increased in A549 cells after rgDV2-NS1-K272R infection (Fig. 7D, E). We further examined the pro-inflammatory cytokine expression in THP-1-derived M1 macrophages stimulated with supernatants from rgDENV-infected A549 cells. The result also showed that elevated IL-6 and IL-8 may be enhanced by soluble NS1 from rgDENV2-NS1-K272R infected cells (Fig. S4).

## Discussion

Four amino acid substitutions were identified in the NS1 region of the 2015 Taiwan outbreak strains. In the growth kinetics experiments, we found that the NS1-K272R mutant virus replicated faster than WT virus and the other mutant viruses in the IFN-I producing cells. Furthermore, we revealed that the K272R amino acid substitution enhance the secretion of soluble NS1 in the culture supernatants. Higher level of sNS1 may contribute to the evasion of host antiviral response by suppressing the phosphorylation of STAT1 protein and ISGs expression. On the other hand, the K272R mutant-infected cells also induced more intense phosphorylation of p65 protein, as well as higher secretion of pro-inflammatory cytokines including IL-6 and IL-8.

The mutation rates of RNA viruses are generally much higher than DNA viruses due to the low-fidelity of their RdRps [34]. In addition to environmental and host factors, we urge to find the viral factors which may contribute to the 2015 Taiwan outbreak. A total of 11 amino acid substitutions were found in 22 dengue infection cases in the 2015 Taiwan outbreak by next-generation sequencing and were considered as the special molecular characteristics of 2015 Taiwan outbreak strains (Table S3). Previous studies also indicated that the mutations on the viral genome may alter the viral virulence and increase their resistance to the host immune system which contribute to outbreak occurrences [35, 36]. NS1 plays an essential role in viral replication as well as viral pathogenesis and immunopathogenesis. Our phylogenetic analysis based on the full-length NS1 sequence showed that the NS1 sequence of 2015 Taiwan DENV2 strains were similar to Cosmopolitan genotype reference strains which was consistent with the previous phylogenetic result based on E gene, indicating that the 2015 Taiwan outbreak strains formed a new clade suggesting a clade replacement occurred (Fig. 1) [29, 31].

Additionally, the 2015 Taiwan outbreak strains were also clustered with circulating China strains from 2015 to 2017 indicating that the outbreak may be caused by the imported DENV2-infected cases from China or the 2015 outbreak strains were circulating in both regions (Fig. 2). The Taiwan DENV2 outbreak associated strains from 2014 to 2015 were also previously shown to be clustered with strains isolated from China based on the complete E gene [25]. As previously mentioned, four amino acid substitutions in NS1 region including P73Q, H224N, K272R, and D278E were identified in the 2015 Taiwan outbreak strains when compared to the nucleotide sequence of Taiwan DENV2 isolates before 2015 in GenBank (Table S3). Surprisingly, we also discovered that the DENV2 clinical isolates from the other Asian countries which belongs to the same clade as the 2015 Taiwan outbreak strains only shared the arginine amino acid substitution at position 272, suggesting that this clade may be defined by R272 amino acid in the NS1 region (Fig. 1). 

In the growth kinetics experiments, we found that the NS1-K272R mutant virus showed a higher replication rate than WT virus in IFN-producing cell line, A549 cells, but not in the IFN-I deficient Vero cells (Fig. 3). Therefore, the K272R mutant virus may inhibit the IFN-I antiviral response of host cells. A single mutation NS1-A188V of the Puerto Rico strain PRVABC-59 isolated during the ZIKV epidemics in 2015 inhibits the TANK binding kinase 1 (TBK1)-induced IFN-β promoter activation [37]. Thus, we hypothesized that the K272R mutant virus may inhibit the IFN-I production or its downstream JAK-STAT signaling pathway, and consequently result in the reduction of ISGs expression. We found that the K272R mutant virus-infected cells secreted more sNS1 into culture supernatant than the WT virus-infected cells (Fig. 4A–C). Previous study by Modhiran et al. has revealed that circulating NS1 interacts with TLR4 to induce IL-6 production [38]. To further clarify the role of soluble NS1 on cytokines secretion, we have detected the differences in the IL-6 and IL-8 levels from M1 macrophage after stimulation by the supernatant collected from rgDENV2-NS1-K272R infected A549 cells (Fig. S4). The result anchors the conclusion that elevated IL-6 and IL-8 may be enhanced by soluble NS1 from rgDENV2-NS1-K272R infected cells. Additionally, we have evaluated the expression of soluble NS1 in rgDENV2-NS1-K272R infections at MOIs of 0.01 and 0.1, observing overall increased levels in NS1 (Fig. 4). Gene expression analysis showed a dose-dependent reduction in* IFIT1* and *MxA* levels, while* ISG15* expression was significantly decreased at both MOIs of 0.01 and 0.1 (Fig. S1). Additionally, IL-6 and IL-8 secretion increased significantly in a dose-dependent manner, starting from day 1 and day 3, respectively, following rgDENV2-NS1-K272R infection (Fig. S3). Since secreted soluble NS1 can be recognized by immune cells through TLR4, leading to the activation of NF-κB and large amounts of pro-inflammatory cytokines being released which may lead to tissue damage and organ failure [8]. Therefore, we suggest that the decreased ISGs expression and higher secretion of pro-inflammatory cytokine induced by rgDV2-NS1-K272R may be due to the higher secretion of soluble NS1.

In a previous study, Ogire et al. found that the lysine to arginine amino acid substitution affects the biological properties of NS1 proteins in Huh7 cells [33]. They found that the recombinant NS1 with arginine at both the position 272 and 324 (rNS1-(R272, R324)) has a higher extracellular protein expression than NS1 with lysine amino acid substitution at position 272 (rNS1-(K272, R324)) which is consistent with our finding. However, the rNS1-(K272, R324) has a lower intracellular expression in Huh7 cells when compared with rNS1-(K272, R324) due to the instability of rNS1-(K272, R324) [33]. In contrast, the rNS1-(K272, R324) can be expressed at an equivalent level with rNS1-(R272, R324) in 293 T cells in our study (Fig. 6D). Furthermore, we also confirmed that the NS1-(R272, R324) was secreted more intensely in the rg virus infection system (Fig. 4A, B). Hence, the NS1 protein with arginine at position 272 has higher extracellular expression and secretion, which may lead to immune activation and increase the risk of disease outcome.

The IFN-β concentration in the culture supernatants showed significantly higher levels at later time points in both K272R mutant virus-infected cells and WT virus-infected cells, thus, suggesting that rgDV2-NS1-K272R did not inhibit the IFN-β production (Fig. 5A–C). To confirm that the ISGs were inhibited by rgDV2-NS1-K272R, we treated the cells with recombinant IFN-α after infection. Interestingly, we found significant higher inhibition of ISGs expression by the K272R mutant virus than WT virus (Fig. 6A–C). These results suggest that the K272R amino acid substitution may enhance the ability of DENV to suppress the ISGs expression. To confirm whether the K272R amino acid substitution influences the ability of NS1 in ISGs expression, we expressed equivalent amount of NS1-WT and NS1-K272R protein by transfection of plasmids overexpressing NS1 into the 293 T cells, significant more reduction of ISGs expression by NS1-K272R than NS1-WT indicating that NS1-K272R showed stronger inhibition of ISGs expression compared to NS1-WT (Fig. 6D–H). We also found that NS1-K272R hijacked the JAK-STAT signaling pathway to suppress the ISGs expression by reducing the STAT1 phosphorylation in both the rg virus infection and pTT5-NS1 plasmid transfection systems (Fig. 6I, J). These results suggest that the DENV NS1 had the ability to inhibit the ISGs expression by hijacking the JAK-STAT signaling pathway and the K272R amino acid substitution enhanced the ISGs inhibition ability of NS1 protein. Kao et al. recently discovered that the DENV NS1 and NS3 directly binds to the integrin-linked kinase (ILK) which induces the elevated suppressor of cytokine signaling 3 (SOCS3) expression inhibiting the phosphorylation of STAT1/2 and expression of ISGs [39]. Our results were consistent with the previous studies and changed upon a single K272R amino acid substitution in the NS1 region which suppressed the host cell IFN-I response by increasing the SOCS3 expression to accelerate DENV replication in the infected cells (Fig. 6K).

In a previous study, cytokine storm has been reported in DF and DHF patients compared to healthy controls in 2015 Taiwan dengue outbreak [29]. The levels of IL-6, IL-1β, IL-4, IL-13, IFN-γ, and granulocyte macrophage colony-stimulating factor (GM-CSF) were significantly increased in the sera of DHF patients compared to DF patients. All the inflammatory cytokines and chemokines including IL-6, IL-1β, IL-4, IL-5, IL-10, IL-13, IFN-γ, TNF-α, GM-CSF, monocyte chemoattractant protein-1 (MCP-1), and macrophage inflammatory protein-1β (MIP-1β) were upregulated in both DF and DHF patients [29]. Among the pro-inflammatory cytokines, IL-6 and IL-8 are elevated significantly during DENV infection [40–42]. In another study, the concentrations of IL-6 in the sera of infected patients reached the peak at the first 6 h after hospitalization and decreased during the 7-days study period. The levels of IL-8 were lower than IL-6 at the early phase but decreased more slowly than IL-6 during the observation period [43]. Additionally, the levels of IL-6 elevated at 3 to 5 days post-symptoms, while the IL-8 expression upregulated at 5 to 6 days post-symptoms in another study [44]. In this study, we observed that the K272R mutant virus triggered higher levels of IL-6 and IL-8 compared to WT virus (Fig. 7). In addition, the higher levels of IL-8 were secreted at a delayed time point compared to IL-6 which was consistent with the previous studies in patients. Hence, the K272R amino acid substitution in NS1 indeed increase the pro-inflammatory cytokines secretion after 2015 TW DENV2 infection.

## Conclusions

**Fig. 8:**
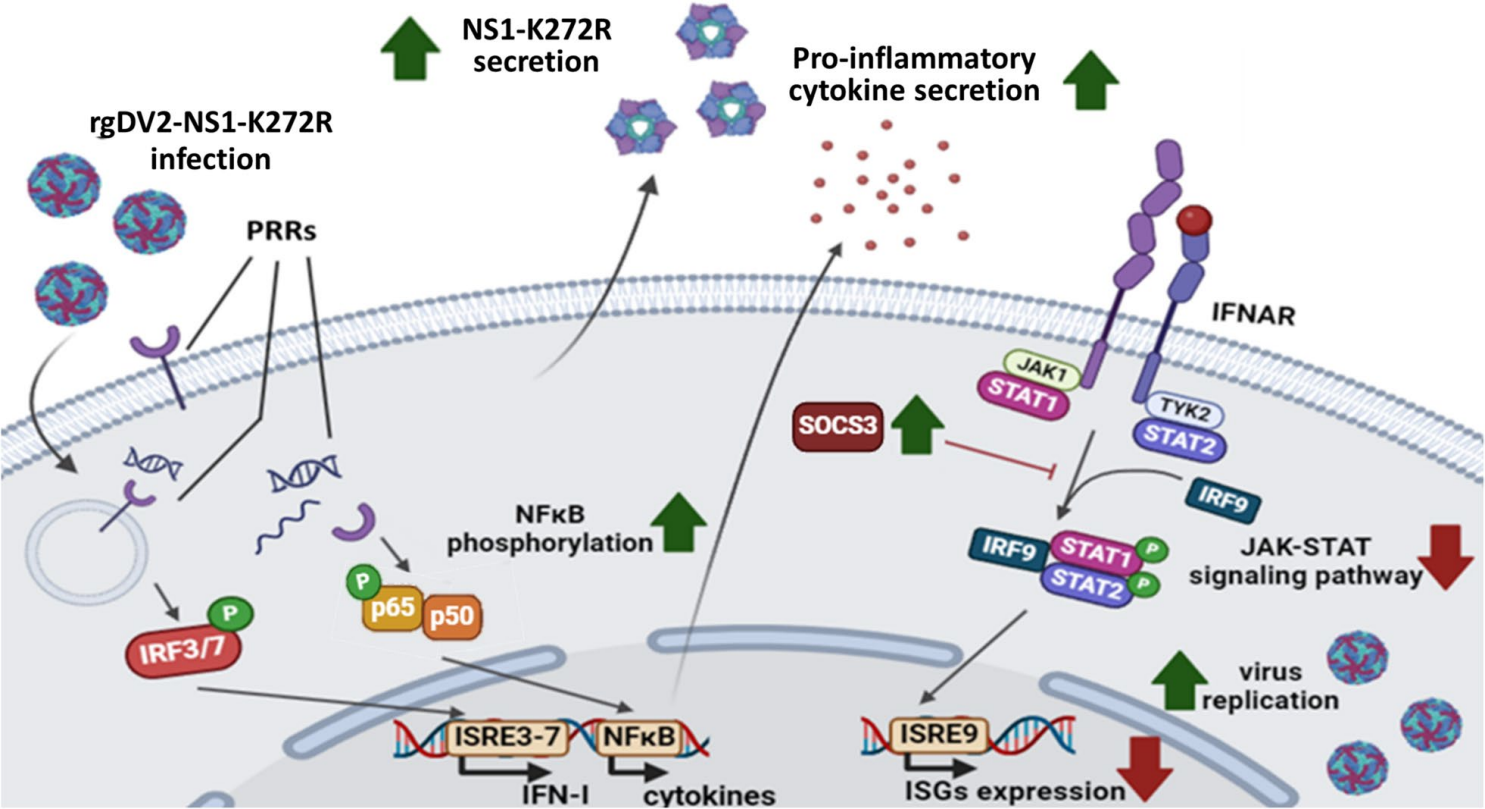
Schematic diagram of effects of K272R amino acid substitution on the NS1 properties upon viral infection. During K272R mutant virus infection, the NS1-K272R inhibited the phosphorylation of STAT1 protein and downstream expression of ISGs to enhance the K272R mutant replication. Besides, the infection of K272R mutant virus induced more intense phosphorylation of p65 and higher secretion of pro-inflammatory cytokines which may be due to the higher production of sNS1

In conclusions, we found that NS1-K272R had a stronger ability to inhibit the expression of ISGs by suppressing the phosphorylation of STAT1 proteins which can accelerate the DENV replication. On the other hand, infection by K272R mutant virus also induced higher pro-inflammatory cytokine production at both the protein and mRNA levels compared to the WT virus, which may be due to the higher secretion of sNS1. A proposed model is shown in Fig. 8. In this study, we provided viral factors which may contribute to the severe 2015 Taiwan dengue outbreak. Continuous surveillance of the DENV evolution contributes to a deeper understanding of global dengue transmission dynamics and the control of epidemics. Besides, understanding the molecular basis of the NS1 region in dengue outbreak strains on virus properties provides strong evidence that this protein may be the appropriate target of antiviral therapy.

## Supplementary Information

Supplementary Material 1: Table S1. List of primers used in the construction of infectious cDNA clones and recombinant NS1 clones. Table S2. List of primers used in quantitative real-time PCR. Table S3. Amino acid sequence variations of Taiwan DENV2 strains from 1995 to 2015. Table S4. Comparison of amino acid sequences of DENV2 strains of Asian countries from 1995 to 2019.

Supplementary Material 2: Fig. S1. K272R amino acid substitution contributed to immune evasion by inhibition of ISGs expression. (A-C) A549 cells were infected with K272R mutant virus at MOI 0.1 and 0.01 for 72 hrs followed by IFN-α treatment (1000 IU/mL) for 6 hrs. Quantitative RT-PCR analysis of ISGs (A) *IFIT1*, (B) *ISG15*, and (C) *MxA* were performed. All data are representative data from at least two independent experiments with ****p < 0.0001, ***p < 0.001, **p < 0.01, *p < 0.05 by one-way ANOVA.

Supplementary Material 3: Fig. S2. rgDENV2-NS1-K272R infection led to ISGs down-regulation in M1 macrophage. THP-1 derived M1 type macrophage cells were infected with K272R mutant virus or WT virus at MOI 0.1 for the indicated time points. Quantitative RT-PCR analysis of ISGs (A)* IFIT1*, (B) *ISG15*, and (C) *MxA* were performed. All data are representative data from at least two independent experiments with ****p < 0.0001, ***p < 0.001, **p < 0.01, *p < 0.05 by one-way ANOVA.

Supplementary Material 4: Fig. S3. K272R mutant virus stimulates pro-inflammatory cytokines expression following the dose-dependent manner. A549 cells were infected with K272R mutant virus at MOI 0.1 and 0.01 for the indicated time points. The secretion level of both (A) IL-6 and (B) IL-8 pro-inflammatory cytokines in culture supernatants were measured by ELISA. All data are representative data from at least two independent experiments with ****p < 0.0001 ***p < 0.001, **p < 0.01 and *p < 0.05 by two-way ANOVA.

Supplementary Material 5: Fig. S4. Increased pro-inflammatory cytokines expression after stimulation by the rgDENV2-NS1-K272R infected A549 cells supernatant. The A549 cells were first infected with K272R mutant or WT virus at MOI 0.1 for 72 hrs. The culture supernatant was further utilized for THP-1 derived macrophage stimulation for 1 and 3 days. The concentration of both (A) IL-6 and (B) IL-8 in culture supernatants were measured by ELISA. All data are representative data from at least two independent experiments with ****p < 0.0001, ***p < 0.001, **p < 0.01, *p < 0.05, and ns, p > 0.05 by two-way ANOVA.

## Data Availability

All DENV2 genomes generated during this study are publicly available in GenBank.

## Abbreviations

DENV: Dengue virus
rg virus: Reverse genetic virus
IFN: Interferon
ISGs: Interferon-stimulated genes
WT: Wildtype
NF-κB: Nuclear factor-kappa B
NS: Non-structural protein
TNF-α: Tumor necrosis factor alpha
MOI: Multiplicity of infection
