# Supplementary material for: A non-structural protein 1 substitution of dengue virus enhances viral replication by interfering with the antiviral signaling pathway
Source: J Biomed Sci. 2025 Feb 20;32:25. doi: 10.1186/s12929-024-01116-4 (PMC11841148; doi:10.1186/s12929-024-01116-4)
Supplement: Supplementary file 1 — Supplementary Material 1: Table S1. List of primers used in the construction of infectious cDNA clones and recombinant NS1 clones. Table S2. List of primers used in quantitative real-time PCR. Table S3. Amino acid sequence variations of Taiwan DENV2 strains from 1995 to 2015. Table S4. Comparison of amino acid sequences of DENV2 strains of Asian countries from 1995 to 2019. [file 12929_2024_1116_MOESM1_ESM.docx]

Table S1. List of primers used in the construction of infectious cDNA clones and recombinant NS1 clones

| **Primer name** | **Sequence (5'-3')** |
| --- | --- |
| pDV2-Scal Fw | AGCCTGCCACCCTAAGGAAGTACTGTATAGAGGC |
| pDV2-Narl Rv | TCCGTCATAGTGGCGCCTACCATAACCAT |
| pDV2-C2543A Fw | TGGAAACAAATAACACAAGAATTGAATCACATTCTATCAG |
| pDV2-C2543A Rv | CTGATAGAATGTGATTCAATTCTTGTGTTATTTGTTTCA |
| pDV2-C2995A Fw | GAAGTTAAAAACTGCAACTGGCCAAAATCACAC |
| pDV2-C2995A Rv | GTGTGATTTTGGCCAGTTGCAGTTTTTAACTTC |
| pDV2-A3140G Fw | GACCATGGCATCTAGGTAGGCTTGAGATGGACTT |
| pDV2-A3140G Rv | AAGTCCATCTCAAGCCTACCTAGATGCCATGGTC |
| pDV2-T3159A Fw | TGAGATGGACTTTGAATTCTGTGATGGAACAACAG |
| pDV2-T3159A Rv | CTGTTGTTCCATCCAGAATTCAAAGTCCATCTCA |
| pNsiI-6His-NS1-1F | ATGCATCATCACCATCACCATCACGATAGTGGTTGCGTT |
| P1056r-NS1-6His-XbaI | TCTAGATTAGTGATGGTGATGGTGATGAGCTGTGACCAAGGA |

Table S2. List of primers used in quantitative real-time PCR

| **Target gene** | **Primer name** | **Sequence (5'-3')** |
| --- | --- | --- |
| IFN-β | IFN-β Fw | CGCCGCATTGACCATCTA |
|  | IFN-β Rv | GACATTAGCCAGGAGGTTCTCA |
| IFIT1 | IFIT1 Fw | TCAGGTCAAGGATAGTCTGGAG |
|  | IFIT1 Rv | AGGTTGTGTATTCCCACACTGTA |
| ISG15 | ISG15 Fw | TCCTGGTGAGGAATAACAAGGG |
|  | ISG15 Rv | GTCAGCCAGAACAGGTCGTC |
| MxA | MxA Fw | ACCACAGAGGCTCTCAGCAT |
|  | MxA Rv | CTCAGCTGGTCCTGGATCTC |
| IL-6 | IL-6 Fw | AGACAGCCACTCACCTCTTCAG |
|  | IL-6 Rv | TTCTGCCAGTGCCTCTTTGCTG |
| IL-8 | IL-8 Fw | ACTGAGAGTGATTGAGAGTGAC |
|  | IL-8 Rv | AACCCTCTGCACCCAGTTTTC |

Table S3. Amino acid sequence variations of Taiwan DENV2 strains from 1995 to 2015

| **DENV2 strain^a^** | **C** | **NS1** | | | | **NS2A** | | | | **NS3** | **NS5** |
| --- | --- | --- | --- | --- | --- | --- | --- | --- | --- | --- | --- |
|  | **73^b^** | **73** | **224** | **272** | **278** | **39** | **104** | **166** | **171** | **395** | **387** |
| **D2/TW/KY670633/1995** | **K** | **P** | **H** | **K** | **D** | **S** | **T** | **K** | **I** | **V** | **K** |
| **D2/TW/MG599594/2001** | **K** | **P** | **H** | **K** | **D** | **S** | **T** | **K** | **I** | **V** | **K** |
| **D2/TW/MG599600/2001** | **K** | **P** | **H** | **K** | **D** | **S** | **T** | **K** | **I** | **V** | **K** |
| **D2/TW/MG599601/2001** | **K** | **P** | **H** | **K** | **D** | **S** | **T** | **K** | **I** | **V** | **K** |
| **D2/TW/MG599606/2001** | **K** | **P** | **H** | **K** | **D** | **S** | **T** | **K** | **I** | **V** | **K** |
| **D2/TW/DQ645541/2001** | **K** | **P** | **H** | **K** | **D** | **S** | **T** | **K** | **I** | **V** | **K** |
| **D2/TW/DQ645542/2001** | **K** | **P** | **H** | **K** | **D** | **S** | **T** | **K** | **I** | **V** | **K** |
| **D2/TW/MG599605/2002** | **K** | **P** | **H** | **K** | **D** | **S** | **T** | **K** | **I** | **V** | **K** |
| **D2/TW/MG599622/2002** | **K** | **P** | **H** | **K** | **D** | **S** | **T** | **K** | **I** | **V** | **K** |
| **D2/TW/MG599619/2002** | **K** | **P** | **H** | **K** | **D** | **S** | **T** | **K** | **I** | **V** | **K** |
| **D2/TW/MG599626/2002** | **K** | **P** | **H** | **K** | **D** | **S** | **T** | **K** | **I** | **V** | **K** |
| **D2/TW/MG599631/2002** | **K** | **P** | **H** | **K** | **D** | **S** | **T** | **K** | **I** | **V** | **K** |
| **D2/TW/MG599628/2002** | **K** | **P** | **H** | **K** | **D** | **S** | **T** | **K** | **I** | **V** | **K** |
| **D2/TW/MG599634/2002** | **K** | **P** | **H** | **K** | **D** | **S** | **T** | **K** | **I** | **V** | **K** |
| **D2/TW/DQ645545/2002** | **K** | **P** | **H** | **K** | **D** | **S** | **T** | **K** | **I** | **V** | **K** |
| **D2/TW/DQ645546/2002** | **K** | **P** | **H** | **K** | **D** | **S** | **T** | **K** | **I** | **V** | **K** |
| **D2/TW/DQ645547/2002** | **K** | **P** | **H** | **K** | **D** | **S** | **T** | **K** | **I** | **V** | **K** |
| **D2/TW/DQ645549/2002** | **K** | **P** | **H** | **K** | **D** | **S** | **T** | **K** | **I** | **V** | **K** |
| **D2/TW/DQ645550/2002** | **K** | **P** | **H** | **K** | **D** | **S** | **T** | **K** | **I** | **V** | **K** |
| **D2/TW/DQ645552/2002** | **K** | **P** | **H** | **K** | **D** | **S** | **T** | **K** | **I** | **V** | **K** |
| **D2/TW/DQ645554/2002** | **K** | **P** | **H** | **K** | **D** | **S** | **T** | **K** | **I** | **V** | **K** |
| **D2/TW/DQ645555/2002** | **K** | **P** | **H** | **K** | **D** | **S** | **T** | **K** | **I** | **V** | **K** |
| **D2/TW/DQ645556/2002** | **K** | **P** | **H** | **K** | **D** | **S** | **T** | **K** | **I** | **V** | **K** |
| **D2/TW/HQ891023/2008** | **K** | **P** | **H** | **K** | **D** | **S** | **T** | **K** | **I** | **V** | **K** |
| **D2/TW/HQ891024/2008** | **K** | **P** | **H** | **K** | **D** | **S** | **T** | **K** | **I** | **V** | **K** |
| **D2/TW/KJ734727/2014** | **K** | **P** | **H** | **K** | **D** | **S** | **T** | **K** | **I** | **V** | **K** |
| **D2/TW/AJ968413/2014** | **K** | **P** | **H** | **K** | **D** | **S** | **T** | **K** | **I** | **V** | **K** |
| **D2/TW/OR593362/2015** | **R** | **Q** | **N** | **R** | **E** | **T** | **A** | **R** | **T** | **I** | **R** |
| **D2/TW/OR593363/2015** | **R** | **Q** | **N** | **R** | **E** | **T** | **A** | **R** | **T** | **I** | **R** |
| **D2/TW/OR593364/2015** | **R** | **Q** | **N** | **R** | **E** | **T** | **A** | **R** | **T** | **I** | **R** |
| **D2/TW/OR593365/2015** | **R** | **Q** | **N** | **R** | **E** | **T** | **A** | **R** | **T** | **I** | **R** |
| **D2/TW/OR593366/2015** | **R** | **Q** | **N** | **R** | **E** | **T** | **A** | **R** | **T** | **I** | **R** |
| **D2/TW/OR593367/2015** | **R** | **Q** | **N** | **R** | **E** | **T** | **A** | **R** | **T** | **I** | **R** |
| **D2/TW/OR593368/2015** | **R** | **Q** | **N** | **R** | **E** | **T** | **A** | **R** | **T** | **I** | **R** |
| **D2/TW/OR593369/2015** | **R** | **Q** | **N** | **R** | **E** | **T** | **A** | **R** | **T** | **I** | **R** |
| **D2/TW/OR593370/2015** | **R** | **Q** | **N** | **R** | **E** | **T** | **A** | **R** | **T** | **I** | **R** |
| **D2/TW/OR593371/2015** | **R** | **Q** | **N** | **R** | **E** | **T** | **A** | **R** | **T** | **I** | **R** |
| **D2/TW/OR593372/2015** | **R** | **Q** | **N** | **R** | **E** | **T** | **A** | **R** | **T** | **I** | **R** |
| **D2/TW/OR593373/2015** | **R** | **Q** | **N** | **R** | **E** | **T** | **A** | **R** | **T** | **I** | **R** |
| **D2/TW/OR593374/2015** | **R** | **Q** | **N** | **R** | **E** | **T** | **A** | **R** | **T** | **I** | **R** |
| **D2/TW/OR593375/2015** | **R** | **Q** | **N** | **R** | **E** | **T** | **A** | **R** | **T** | **I** | **R** |
| **D2/TW/OR593376/2015** | **R** | **Q** | **N** | **R** | **E** | **T** | **A** | **R** | **T** | **I** | **R** |
| **D2/TW/OR593377/2015** | **R** | **Q** | **N** | **R** | **E** | **T** | **A** | **R** | **T** | **I** | **R** |
| **D2/TW/OR593378/2015** | **R** | **Q** | **N** | **R** | **E** | **T** | **A** | **R** | **T** | **I** | **R** |
| **D2/TW/OR593379/2015** | **R** | **Q** | **N** | **R** | **E** | **T** | **A** | **R** | **T** | **I** | **R** |
| **D2/TW/OR593380/2015** | **R** | **Q** | **N** | **R** | **E** | **T** | **A** | **R** | **T** | **I** | **R** |
| **D2/TW/OR593381/2015** | **R** | **Q** | **N** | **R** | **E** | **T** | **A** | **R** | **T** | **I** | **R** |
| **D2/TW/OR593382/2015** | **R** | **Q** | **N** | **R** | **E** | **T** | **A** | **R** | **T** | **I** | **R** |
| **D2/TW/OR593383/2015** | **R** | **Q** | **N** | **R** | **E** | **T** | **A** | **R** | **T** | **I** | **R** |

^a^The sequences of DENV2 Taiwan strains from 1995-2014 collected from GenBank were marked in blue while the 2015 DENV2 outbreak strains sequenced with the Illumina Miseq platform were marked in red. TW: Taiwan.

^b^The amino acids identical to TW DENV2 strains from 1995 to 2014 were marked in blue while the amino acids consistent with 2015 TW outbreak strains were marked in red.

Table S4. Comparison of amino acid sequences of DENV2 strains of Asian countries from 1995 to 2019

| **DENV2 strain^a,b^** | **C** | **NS1** | | | | **NS2A** | | | | **NS3** | **NS5** |
| --- | --- | --- | --- | --- | --- | --- | --- | --- | --- | --- | --- |
|  | **73^c^** | **73** | **224** | **272** | **278** | **39** | **104** | **166** | **171** | **395** | **387** |
| **D2/SG/JN851123/2004** | **K** | **P** | **H** | **R** | **D** | **S** | **T** | **K** | **T** | **I** | **K** |
| **D2/SG/JN851130/2005** | **K** | **P** | **Y** | **R** | **D** | **S** | **T** | **K** | **T** | **I** | **K** |
| **D2/VN/EU482640/2006** | **K** | **P** | **H** | **R** | **D** | **S** | **T** | **K** | **T** | **I** | **K** |
| **D2/ID/KC762655/2007** | **K** | **P** | **H** | **R** | **D** | **S** | **T** | **K** | **T** | **I** | **K** |
| **D2/ID/KC762662/2007** | **K** | **P** | **H** | **R** | **D** | **S** | **T** | **K** | **T** | **I** | **K** |
| **D2/SG/JN851114/2007** | **K** | **P** | **H** | **R** | **D** | **S** | **T** | **K** | **T** | **I** | **K** |
| **D2/ID/KC762663/2008** | **K** | **P** | **H** | **R** | **D** | **S** | **T** | **K** | **T** | **I** | **K** |
| **D2/ID/KC762672/2008** | **K** | **P** | **H** | **R** | **D** | **S** | **T** | **K** | **T** | **I** | **K** |
| **D2/IN/KC762666/2008** | **K** | **P** | **H** | **R** | **D** | **S** | **T** | **K** | **T** | **I** | **K** |
| **D2/ID/KU509268/2009** | **K** | **P** | **H** | **R** | **D** | **S** | **T** | **K** | **T** | **I** | **K** |
| **D2/SG/JF327392/2009** | **K** | **P** | **H** | **R** | **D** | **S** | **T** | **K** | **T** | **I** | **K** |
| **D2/SG/KM279609/2009** | **K** | **P** | **H** | **R** | **D** | **S** | **T** | **K** | **T** | **I** | **K** |
| **D2/TH/KU509272/2009** | **K** | **P** | **H** | **R** | **D** | **S** | **T** | **K** | **T** | **I** | **K** |
| **D2/CN/KP723479/2010** | **K** | **P** | **H** | **R** | **D** | **S** | **T** | **K** | **T** | **I** | **K** |
| **D2/ID/KC762678/2010** | **K** | **P** | **H** | **R** | **D** | **S** | **T** | **K** | **T** | **I** | **K** |
| **D2/ID/KC762680/2010** | **K** | **P** | **H** | **R** | **D** | **S** | **T** | **K** | **T** | **I** | **K** |
| **D2/IN/KC762679/2010** | **K** | **P** | **H** | **R** | **D** | **S** | **T** | **K** | **T** | **I** | **K** |
| **D2/SG/KX380807/2012** | **K** | **P** | **H** | **R** | **D** | **S** | **T** | **K** | **I** | **I** | **K** |
| **D2/SG/KX380815/2012** | **K** | **P** | **H** | **R** | **D** | **S** | **T** | **K** | **T** | **V** | **K** |
| **D2/SG/KX380832/2013** | **K** | **P** | **H** | **R** | **D** | **S** | **T** | **K** | **I** | **I** | **K** |
| **D2/CN/KT187554/2014** | **K** | **P** | **H** | **R** | **D** | **S** | **T** | **K** | **T** | **I** | **K** |
| **D2/ID/MH823208/2014** | **K** | **P** | **H** | **R** | **D** | **S** | **T** | **K** | **I** | **I** | **K** |
| **D2/MY/KU666944/2014** | **K** | **P** | **H** | **R** | **D** | **S** | **T** | **K** | **A** | **V** | **K** |
| **D2/MY/KU666949/2014** | **K** | **P** | **H** | **R** | **D** | **S** | **T** | **K** | **T** | **I** | **K** |
| **D2/CN/KX225485/2015** | **K** | **P** | **H** | **R** | **D** | **S** | **T** | **K** | **T** | **I** | **K** |
| **D2/CN/KX621248/2015** | **K** | **P** | **H** | **R** | **D** | **S** | **T** | **K** | **A** | **V** | **K** |
| **D2/KR/MK629884/2015** | **K** | **P** | **H** | **R** | **D** | **S** | **T** | **K** | **A** | **V** | **K** |
| **D2/PH/KU517847/2015** | **K** | **P** | **H** | **R** | **D** | **S** | **T** | **K** | **A** | **V** | **K** |
| **D2/SG/MK513444/2015** | **K** | **P** | **H** | **R** | **D** | **S** | **T** | **K** | **A** | **V** | **K** |
| **D2/CN/MN018352/2015** | **R** | **Q** | **N** | **R** | **E** | **T** | **A** | **R** | **T** | **I** | **R** |
| **D2/TW/OR593362/2015** | **R** | **Q** | **N** | **R** | **E** | **T** | **A** | **R** | **T** | **I** | **R** |
| **D2/TW/OR593363/2015** | **R** | **Q** | **N** | **R** | **E** | **T** | **A** | **R** | **T** | **I** | **R** |
| **D2/TW/OR593364/2015** | **R** | **Q** | **N** | **R** | **E** | **T** | **A** | **R** | **T** | **I** | **R** |
| **D2/TW/OR593365/2015** | **R** | **Q** | **N** | **R** | **E** | **T** | **A** | **R** | **T** | **I** | **R** |
| **D2/TW/OR593366/2015** | **R** | **Q** | **N** | **R** | **E** | **T** | **A** | **R** | **T** | **I** | **R** |
| **D2/TW/OR593367/2015** | **R** | **Q** | **N** | **R** | **E** | **T** | **A** | **R** | **T** | **I** | **R** |
| **D2/TW/OR593368/2015** | **R** | **Q** | **N** | **R** | **E** | **T** | **A** | **R** | **T** | **I** | **R** |
| **D2/TW/OR593369/2015** | **R** | **Q** | **N** | **R** | **E** | **T** | **A** | **R** | **T** | **I** | **R** |
| **D2/TW/OR593370/2015** | **R** | **Q** | **N** | **R** | **E** | **T** | **A** | **R** | **T** | **I** | **R** |
| **D2/TW/OR593371/2015** | **R** | **Q** | **N** | **R** | **E** | **T** | **A** | **R** | **T** | **I** | **R** |
| **D2/TW/OR593372/2015** | **R** | **Q** | **N** | **R** | **E** | **T** | **A** | **R** | **T** | **I** | **R** |
| **D2/TW/OR593373/2015** | **R** | **Q** | **N** | **R** | **E** | **T** | **A** | **R** | **T** | **I** | **R** |
| **D2/TW/OR593374/2015** | **R** | **Q** | **N** | **R** | **E** | **T** | **A** | **R** | **T** | **I** | **R** |
| **D2/TW/OR593375/2015** | **R** | **Q** | **N** | **R** | **E** | **T** | **A** | **R** | **T** | **I** | **R** |
| **D2/TW/OR593376/2015** | **R** | **Q** | **N** | **R** | **E** | **T** | **A** | **R** | **T** | **I** | **R** |
| **D2/TW/OR593377/2015** | **R** | **Q** | **N** | **R** | **E** | **T** | **A** | **R** | **T** | **I** | **R** |
| **D2/TW/OR593378/2015** | **R** | **Q** | **N** | **R** | **E** | **T** | **A** | **R** | **T** | **I** | **R** |
| **D2/TW/OR593379/2015** | **R** | **Q** | **N** | **R** | **E** | **T** | **A** | **R** | **T** | **I** | **R** |
| **D2/TW/OR593380/2015** | **R** | **Q** | **N** | **R** | **E** | **T** | **A** | **R** | **T** | **I** | **R** |
| **D2/TW/OR593381/2015** | **R** | **Q** | **N** | **R** | **E** | **T** | **A** | **R** | **T** | **I** | **R** |
| **D2/TW/OR593382/2015** | **R** | **Q** | **N** | **R** | **E** | **T** | **A** | **R** | **T** | **I** | **R** |
| **D2/TW/OR593383/2015** | **R** | **Q** | **N** | **R** | **E** | **T** | **A** | **R** | **T** | **I** | **R** |
| **D2/CN/MK564477/2016** | **R** | **Q** | **N** | **R** | **E** | **T** | **A** | **R** | **T** | **I** | **R** |
| **D2/CN/MK564478/2016** | **K** | **P** | **H** | **R** | **D** | **S** | **T** | **K** | **I** | **I** | **K** |
| **D2/CN/MN018353/2016** | **K** | **P** | **H** | **R** | **D** | **S** | **T** | **K** | **I** | **I** | **K** |
| **D2/ID/MK411558/2016** | **K** | **P** | **H** | **R** | **D** | **S** | **T** | **K** | **I** | **I** | **K** |
| **D2/SG/MF314189/2016** | **K** | **P** | **H** | **R** | **D** | **S** | **T** | **K** | **A** | **V** | **K** |
| **D2/TH/LC410190/2016** | **K** | **P** | **H** | **R** | **D** | **S** | **T** | **K** | **A** | **V** | **K** |
| **D2/CN/MH010629/2017** | **K** | **P** | **H** | **R** | **D** | **S** | **T** | **K** | **A** | **V** | **K** |
| **D2/CN/MK564482/2017** | **R** | **Q** | **N** | **R** | **E** | **T** | **A** | **R** | **T** | **I** | **R** |
| **D2/CN/MK564486/2018** | **K** | **P** | **H** | **R** | **D** | **S** | **T** | **K** | **T** | **I** | **K** |
| **D2/BD/MN328061/2019** | **K** | **P** | **H** | **R** | **D** | **S** | **T** | **K** | **A** | **V** | **K** |
| **D2/CN/MN923112/2019** | **K** | **P** | **H** | **R** | **D** | **S** | **T** | **K** | **I** | **I** | **K** |
| **D2/CN/MN923116/2019** | **K** | **P** | **H** | **R** | **D** | **S** | **T** | **K** | **I** | **I** | **K** |

^a^The sequences of DENV2 strains of other Asian countries from 1995-2019 were collected from GenBank while the 2015 DENV2 outbreak strains were sequenced with the Illumina Miseq platform.

^b^TW, Taiwan; CN, China; IN, India; ID, Indonesia; KR, Korea; SG, Singapore; MY, Malaysia; TH, Thailand; VN, Vietnam; PH, Philippines; KH, Cambodia; LA, Laos; BD, Bangladesh.

^c^The amino acids identical to TW DENV2 strains from 1995 to 2014 were marked in blue while the amino acids consistent with 2015 TW outbreak strains were marked in red.
