# Supplementary figures and images for: A non-structural protein 1 substitution of dengue virus enhances viral replication by interfering with the antiviral signaling pathway
Source: J Biomed Sci. 2025 Feb 20;32:25. doi: 10.1186/s12929-024-01116-4 (PMC11841148; doi:10.1186/s12929-024-01116-4)

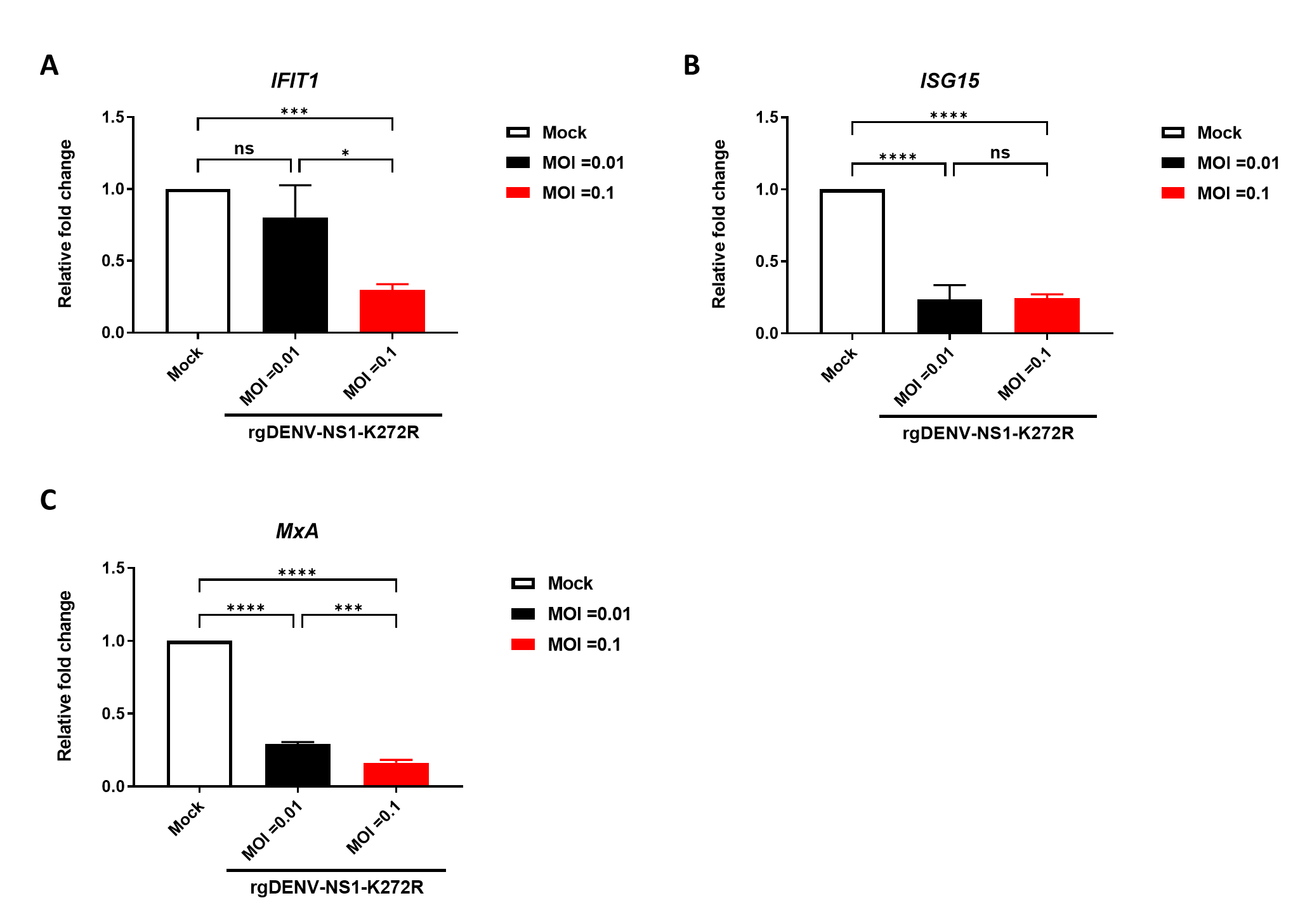

Supplement: Supplementary file 2 — Supplementary Material 2: Fig. S1. K272R amino acid substitution contributed to immune evasion by inhibition of ISGs expression. (A-C) A549 cells were infected with K272R mutant virus at MOI 0.1 and 0.01 for 72 hrs followed by IFN-α treatment (1000 IU/mL) for 6 hrs. Quantitative RT-PCR analysis of ISGs (A) IFIT1, (B) ISG15, and (C) MxA were performed. All data are representative data from at least two independent experiments with ****p < 0.0001, ***p < 0.001, **p < 0.01, *p < 0.05 by one-way ANOVA. [file 12929_2024_1116_MOESM2_ESM.tif]

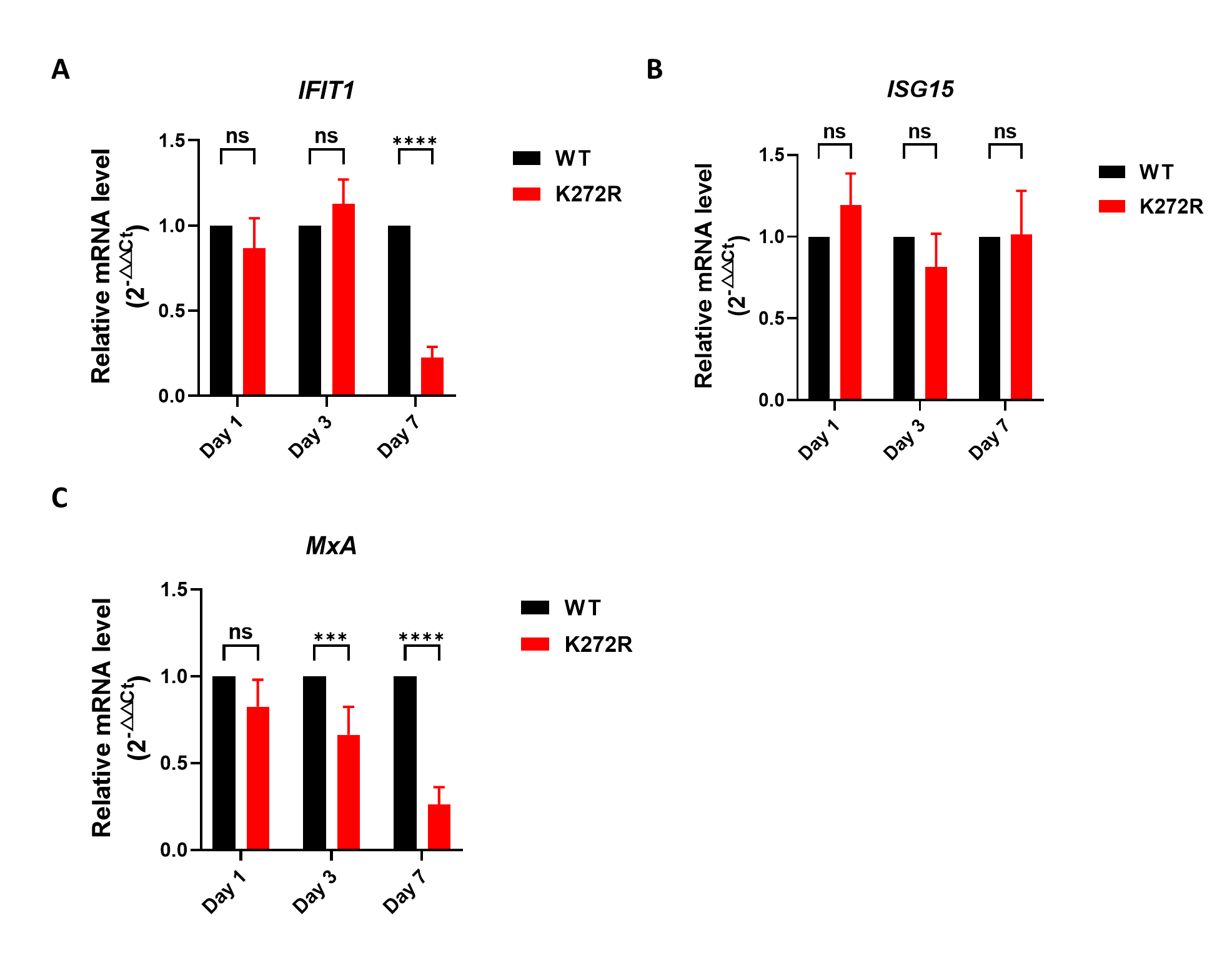

Supplement: Supplementary file 3 — Supplementary Material 3: Fig. S2. rgDENV2-NS1-K272R infection led to ISGs down-regulation in M1 macrophage. THP-1 derived M1 type macrophage cells were infected with K272R mutant virus or WT virus at MOI 0.1 for the indicated time points. Quantitative RT-PCR analysis of ISGs (A) IFIT1, (B) ISG15, and (C) MxA were performed. All data are representative data from at least two independent experiments with ****p < 0.0001, ***p < 0.001, **p < 0.01, *p < 0.05 by one-way ANOVA. [file 12929_2024_1116_MOESM3_ESM.tif]

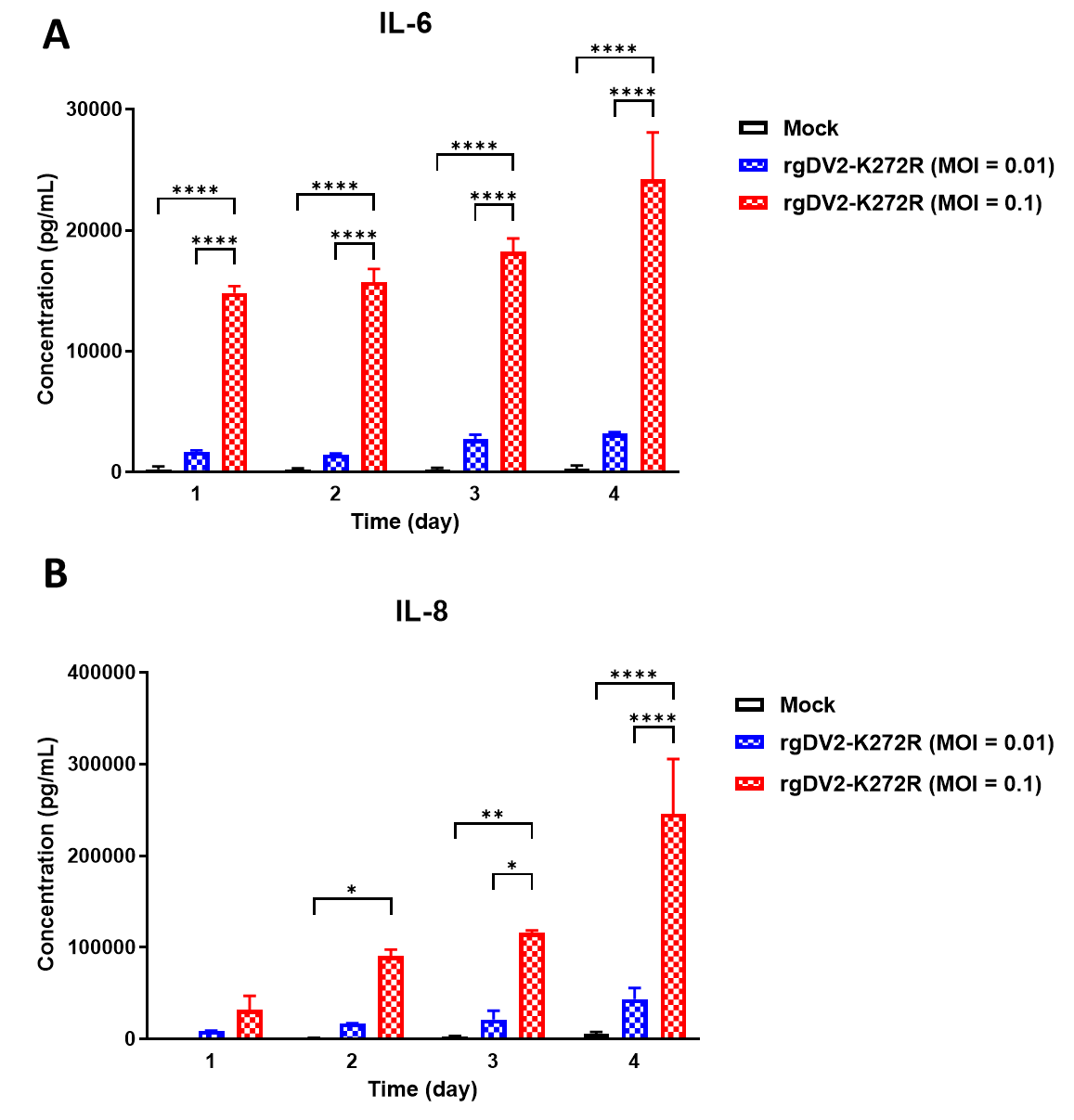

Supplement: Supplementary file 4 — Supplementary Material 4: Fig. S3. K272R mutant virus stimulates pro-inflammatory cytokines expression following the dose-dependent manner. A549 cells were infected with K272R mutant virus at MOI 0.1 and 0.01 for the indicated time points. The secretion level of both (A) IL-6 and (B) IL-8 pro-inflammatory cytokines in culture supernatants were measured by ELISA. All data are representative data from at least two independent experiments with ****p < 0.0001 ***p < 0.001, **p < 0.01 and *p < 0.05 by two-way ANOVA. [file 12929_2024_1116_MOESM4_ESM.tif]

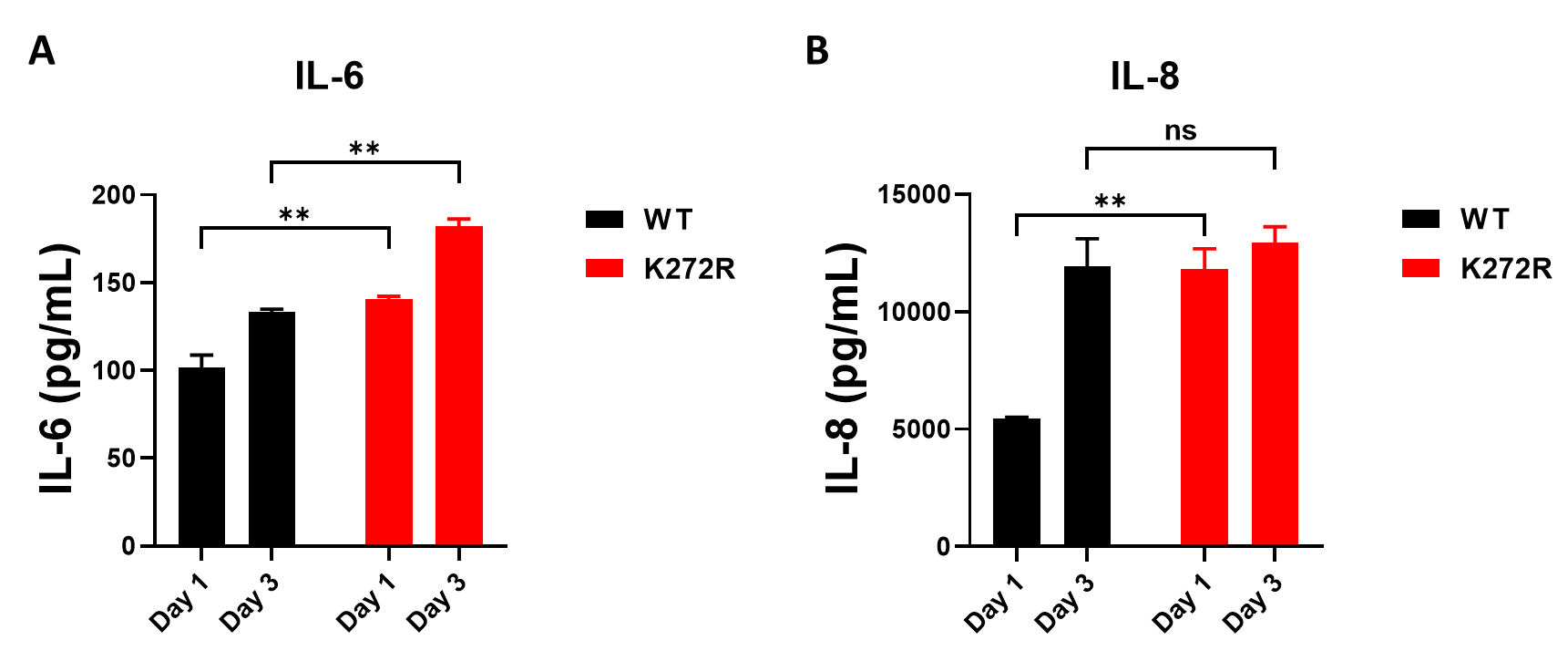

Supplement: Supplementary file 5 — Supplementary Material 5: Fig. S4. Increased pro-inflammatory cytokines expression after stimulation by the rgDENV2-NS1-K272R infected A549 cells supernatant. The A549 cells were first infected with K272R mutant or WT virus at MOI 0.1 for 72 hrs. The culture supernatant was further utilized for THP-1 derived macrophage stimulation for 1 and 3 days. The concentration of both (A) IL-6 and (B) IL-8 in culture supernatants were measured by ELISA. All data are representative data from at least two independent experiments with ****p < 0.0001, ***p < 0.001, **p < 0.01, *p < 0.05, and ns, p > 0.05 by two-way ANOVA. [file 12929_2024_1116_MOESM5_ESM.tif]
